# Synthesis and Validity of Accelerometer Devices and Methods Used in Epidemiological Studies of Physical Activity Bout Duration and Health Outcomes: A Systematic Review

**DOI:** 10.1186/s40798-026-01039-4

**Published:** 2026-07-01

**Authors:** Adrien Chanteau, Germain Faity, Guillaume Mahe, Séverine Sabia, Alexis Le Faucheur

**Affiliations:** 1 M2S (Laboratoire Mouvement, Sport, Santé), Univ Rennes, F-35000 Rennes, France; 2 CIC 1414 (Clinical Investigation Center), CHU Rennes, Inserm, F-35000 Rennes, France; 3 U1153, CRESS, Epidemiology of Ageing and Neurodegenerative Diseases, Université Paris Cité, INSERM, F-75010 Paris, France; 4 Faculty of Brain Sciences, University College London, London, UK

**Keywords:** Physical activity (MeSH), Epidemiology (MeSH), Accelerometry (MeSH), Methods (MeSH), Wearable devices (MeSH)

## Abstract

**Background:**

A paradigm shift in physical activity (PA) research and recommendations emerged in the most recent guidelines, which now emphasize that all physical activity bouts count,irrespective of their duration. Nevertheless, the validity of accelerometer devices and methods for detecting PA bouts of any duration, as considered in studies underpinning the PA guidelines on bout duration and health, remains unclear. The aim of this systematic review was to systematically analyze the validity level of accelerometer devices and methods that have been used in epidemiological studies on the association between PA bout duration and health outcomes.

**Methods:**

This systematic review followed the PRISMA (Preferred Reporting Items for Systematic Reviews and Meta-Analyses) guidelines and employed a two-step search strategy. First, we identified the existing body of evidence on PA bout duration and health by updating the systematic search originally performed for the 2018 Physical Activity Guidelines Advisory Committee scientific report and subsequently expanded by Jakicic et al. Three databases (PubMed, CINAHL, and Cochrane) were searched up to 14 November 2024. The second and final step, which constituted the primary focus of this review, was to retrieve and systematically evaluate the underlying validity studies cited in these epidemiological investigations, with risk of bias assessed using a modified version of the QUADAS-2 tool.

**Results:**

A total of 48 epidemiological studies were included. Analysis of device type, position, epoch duration, drop time, bout duration, and start year of study data collection revealed 113 methodological instances, representing 68 unique combinations, underscoring the heterogeneity in practice. Among the 48 epidemiological studies, 26 validation studies were referenced, and only four were deemed suitable for addressing bout performance detection, accounting for less than 20% of epidemiological studies. For those four studies, the overall study risk of bias was ranked as “High” or with “some concerns”.

**Conclusions:**

Better accuracy and harmonization of accelerometer devices and methods are crucial for broader adoption in national health surveillance systems, ensuring comparability of results and facilitating global knowledge.

**Registration:**

This systematic review was registered on PROSPERO (CRD42023394705).

**Supplementary Information:**

The online version contains supplementary material available at 10.1186/s40798-026-01039-4.

## Key Points

- Epidemiological studies on the relationship between physical activity bout duration and health outcomes demonstrated high heterogeneity in the accelerometric methods and devices used for assessing bout duration.
- Only 15% of validation studies cited in epidemiological research formally assessed the accuracy of accelerometer devices and methods used for physical activity bout detection, accounting for less than 20% of epidemiological studies.
- Further research and consensus on the harmonization of accelerometer methods are needed to establish optimal approaches for detecting and characterizing physical activity bouts.

**Supplementary Information:**

The online version contains supplementary material available at 10.1186/s40798-026-01039-4.

## Background

The accumulated evidence of the benefits of physical activity (PA) for health has formed the foundation for the development of PA recommendations for public health [1, 2]. Previous guidelines acknowledged the health benefits of moderate-to-vigorous physical activity (MVPA) only for bouts lasting at least 10 min [3–5]. However, a paradigm shift in PA research and recommendations emerged in the most recent guidelines, which now emphasize that “all physical activity bout counts”, irrespective of its duration [1, 2]. The removal of the requirement to accumulate physical activity in bouts of at least 10 min does not lessen the importance of quantifying discrete PA bouts. Rather, as emphasized in the 2018 Physical Activity Guidelines Advisory Committee (PAGAC) scientific report, evidence remains insufficient to determine whether the health benefits of PA differ according to bout duration [6]. In this context, the PAGAC highlighted the urgent need for research to establish whether varying bout lengths exert distinct effects on health outcomes, beyond the total volume of accumulated activity irrespective of its pattern.

The shift in recommendations of PA bout duration has important consequences for achieving the recommended amount of PA considering that the human daily PA pattern is highly intermittent [7], with short to very short durations of PA bouts performed at different intensity levels (light, moderate, and vigorous). However, capturing such granular characteristics of PA is challenging. The fact that previous guidelines recognized only the health benefits of MVPA bouts lasting at least 10 min can largely be attributed to the use of questionnaires for PA measurement, which are not meant to capture shorter durations of PA bouts [1, 8]. This paradigm shift in PA recommendations has been driven by the growing use of wearable devices in epidemiological research, enabling a more detailed characterization of PA bouts. In this regard, in the dedicated part concerning the association between PA bout duration and health outcomes of the 2018 PAGAC scientific report [6], 20 out of the 25 included studies used wearable devices, and mostly used accelerometer devices (16 out of 20).

A PA bout can be characterized by its Frequency, Intensity, Time (duration), and Type (e.g., walking), as acknowledged by the FITT principle [9]. A valid assessment of PA bouts first requires accurate detection for then characterizing each of its components. There is extensive literature on the validity of accelerometry for assessing PA intensity or energy expenditure [10]. However, the validity of accelerometer devices and methods for detecting PA bouts of any duration, as considered in studies underpinning the PA guidelines on bout duration and health, remains unclear. This uncertainty questions our ability to accurately detect this information and may explain the difficulties encountered in determining health effects according to the components of PA bouts [6]. It is therefore essential to have a comprehensive understanding of the methods and devices, as well as their validity, that have been used in epidemiological studies that form the foundation of current (and future) PA guidelines. Such understanding would facilitate the identification of the most appropriate methods and devices for PA epidemiology, promote the harmonization of methods, support between-study comparisons and, if needed, highlight the need for the development of new methods, as well as their potential directions.

The aim of this systematic review was to analyze the validity level of accelerometer devices and methods that have been used in epidemiological studies on the association between PA bout duration and health outcomes.

## Methods

This systematic review adhered to the Preferred Reporting Items for Systematic Reviews and Meta-Analyses 2020 statement [11] (supplemental material 1) and was prospectively registered in the PROSPERO database (CRD42023394705). To address the primary aim of this systematic review, we employed a two-step search strategy. In part A, we identified the existing body of evidence regarding PA bout duration and health by updating the systematic search originally conducted for the 2018 PAGAC report and later expanded by Jakicic et al. [12], extending it to include literature published through November 2024. The objective was not to re-examine the available body of evidence in terms of associations between bout duration and health outcomes, but rather to focus on the accelerometer devices and methods used in those epidemiological studies, evaluating their suitability for the intended purpose. Thus, in part B, we retrieved and systematically reviewed the underlying validity studies referenced within those epidemiological investigations.

### Search Strategy

PubMed, CINAHL and Cochrane Library databases were searched up to 14 November 2024 via the search method applied by Jakicic et al. [12]. As initially conducted, separate research was carried out concurrently to identify (S1) original research articles; (S2) systematic reviews, meta-analyses, and pooled analyses. The specific keywords and the full search strategy can be found in supplemental material 2. The following restrictions for article searches were applied: English language and human.

### Eligibility Criteria

To identify the existing body of evidence regarding PA bout duration and health, eligibility criteria were based on those used by Jakicic et al. [12]. However, considering the topic of the present systematic review, only studies using accelerometers or other direct measures to assess bouts of PA as the primary exposure were considered. Furthermore, training/exercise studies were excluded since free-living PA bouts were not studied as an exposure. Studies were included if at least one of the following health outcomes was addressed: all-cause and cardiovascular disease mortality, cardiovascular disease, type 2 diabetes, cardiometabolic risk factors, weight status, body composition, waist circumference, cardiorespiratory fitness, and frailty. Those criteria were applied to the articles selected from both the new search and the systematic review by Jakicic et al. [12]. The full list of inclusion and exclusion criteria can be found in supplemental material 3.

### Selection Process

The selection process was performed via Rayyan software (https://new.rayyan.ai/). This automation tool allows automatic detection and manual removal of duplicate references and then allows blind screening at both the title/abstract and full-text levels. Two authors (ALF and AC) blindly assessed 100% of the titles and abstracts for potential inclusion. In the case of disagreements between reviewers, the paper was retrieved in full text, and mutual consensus was reached. The remaining articles were then screened independently for inclusion at the full-text level by two authors (ALF and AC). Similarly, conflicts were resolved by discussion between the reviewers, and if needed, a consensus was reached with a third author (SS).

During the selection process, systematic reviews and meta-analyses meeting the inclusion criteria were given specific screening. Harmonized meta-analyses were included as independent studies since they provided new data and evidence. To avoid duplicate selection, other systematic reviews and meta-analyses were only checked to identify and add new studies and did not consider previously selected original studies.

### Data Collection Process and Data Items

All data were extracted by one author (AC), and a second author (ALF) verified 100% of the extracted data.

First, from each of the included epidemiological studies, study design, participants, protocol and monitor characteristics, bout definition (see Table 1 for the glossary of bout definition parameters) and underlying cited validation articles were extracted. Validation studies were retrieved either directly from citations provided in the included epidemiological studies or indirectly from studies cited in sources referenced by the included epidemiological studies (citation tracking).

**Table 1:** Characteristics of the epidemiological studies included in the systematic review

Source	Study	Study design	Participants	Protocol	Monitor (type, location, epoch length)	Bout definition (FITT)	Validation article
PAGAC	Ayabe et al., [41]	Cross-sectional	42 healthy participants (42 w, 50 yr)	10-day measurement period	Lifecorder-Ex (uniaxial accelerometer, hip/waist-worn, 4 s)	F: not studied I: LIPA scale 1–3/MPA scale 4–6/VPA scale 7–9/MVPA scale 4–9 (monitor calculated) T: ≥ 32 s / ≥ 1 min / ≥ 3 min / ≥ 5 min / ≥ 10 min T: daily physical activity	Kumahara et al., [100]
PAGAC	Ayabe et al., [42]	Cross-sectional	42 healthy participants (42 w, 50 yr)	10-day measurement period	Lifecorder-Ex (uniaxial accelerometer, hip/waist-worn, 4 s)	F: not studied I: LIPA scale 1–3/MPA scale 4–6/VPA scale 7–9/MVPA scale 4–9 (monitor calculated) T: ≥ 32 s / ≥ 1 min / ≥ 3 min / ≥ 5 min / ≥ 10 min T: daily physical activity	Kumahara et al., [100]
PAGAC	Cameron et al., [43]	Cross-sectional	236 overweight/obese Latino and non-Latino adults (184 w/52 m, 42.6 yr)	7-day measurement period. A valid day consisted of > 10 h of wear time	ActiGraph GT1M and GT3X + (NK, hip/waist-worn, 30 s)	F: not studied I: MVPA ≥ 2020 cpm/VPA ≥ 5999 cpm (categorically calculated with cut-points) T: ≥ 30 s / ≥ 10 min (with drop time: “allowing for an interruption of up to 2 min anywhere within the bout”) T: daily physical activity	Troiano et al., [108]
PAGAC	Clarke and Janssen, [44]	Cross-sectional	1119 participants from CHMS cohort (532 w/587 m, 41.1 yr)	7-day measurement period. A valid day consisted of > 10 h of wear time, with a minimum of 4 valid days	Actical (omnidirectional accelerometer, hip/waist-worn, 60 s)	F: not studied I: MVPA ≥ 1535 cpm (each epoch above the MPA cut-point is qualified using a regression equation to estimate METs) T: ≥ 1 min / ≥ 10 min (with drop time: “a bout continued until 80% was no longer above the cut-point”) T: daily physical activity	Colley and Tremblay, [89]
PAGAC	Di Blasio et al., [66]	Prospective	40 post-menopausal participants (40 w, 56.8 yr)	3-day measurement period (two weekdays and one weekend day)	SenseWear Pro2 (multisensor device with bi-axial accelerometer, upper arm-worn, NK)	F: not studied I: MPA/VPA (monitor calculated) T: ≥ 1epoch^a^ / ≥ 5 min / ≥ 10 min T: daily physical activity	Welk et al., [110]
PAGAC	Fan et al., [45]	Cross-sectional	4511 participants from NHANES 2003–2006 cohort (2202 w/2309 m, 42.7 yr)	7-day measurement period. A valid day consisted of > 10 h of wear time, with a minimum of 4 valid days	Actigraph 7164 (uniaxial accelerometer, hip/waist-worn, 60 s)	F: not studied I: lower-intensity 760–2019 cpm/MVPA ≥ 2020 cpm (categorically calculated with cut-points) T: ≥ 1 min / ≥ 10 min (with drop time: “allowing for 1–2 min interruptions within any 10-min window”) T: daily physical activity	Troiano et al., [108]
PAGAC	Gay et al., [46]	Cross-sectional	5302 participants from NHANES 2003–2006 cohort (2710 w/2592 m, 47 yr)	7-day measurement period. A valid day consisted of > 10 h of wear time, with a minimum of 4 valid days	Actigraph 7164 (uniaxial accelerometer, hip/waist-worn, 60 s)	F: not studied I: MVPA ≥ 2020 cpm (categorically calculated with cut-point) T: ≥ 1 min/ ≥ 10 min T: daily physical activity	Troiano et al., [108]
PAGAC	Glazer et al., [47]	Cross-sectional	2109 participants from Framingham heart study 3rd gen. cohort (1150 w/959 m, 47 yr)	7-day measurement period. A valid day consisted of > 10 h of wear time, with a minimum of 5 valid days	Actical (omnidirectional accelerometer, hip/waist-worn, 30 s)	F: not studied I: MVPA ≥ 1486 cpm (categorically calculated with cut-point using a weighted average of 2 studies) T: ≥ 30 s/ ≥ 10 min (with drop time: “allowing for a 1–2 min interruption”) T: daily physical activity	Crouter and Basset, [91] Heil, [95]
PAGAC	Jefferis et al., [48]	Cross-sectional	1009 participants from British regional heart study cohort (1009 m, 78.5 yr)	7-day measurement period. A valid day consisted of > 10 h of wear time, with a minimum of 3 valid days	Actigraph GT3X (uniaxial accelerometer, hip/waist-worn, 60 s)	F: not studied I: LIPA 100–1040 cpm/MVPA > 1040 cpm (categorically calculated with cut-points) T: 1–9 min / ≥ 10 min T: daily physical activity	Copeland and Esliger, [90]
PAGAC	Kehler et al., [49]	Cross-sectional	2317 participants from NHANES 2003–2006 cohort (1143 w/1174 m, 67.4 yr)	7-day measurement period. A valid day consisted of > 10 h of wear time, with a minimum of 4 valid days	Actigraph 7164 (uniaxial accelerometer, hip/waist-worn, 60 s)	F: not studied I: MVPA ≥ 2020 cpm (categorically calculated with cut-point) T: ≥ 1 min/ ≥ 10 min (with drop time: “with two allowable consecutive minutes out of 10 min to drop below the MVPA intensity threshold into light-intensity physical activity”) T: daily physical activity	Troiano et al., [108]
PAGAC	Loprinzi et al., [51]	Cross-sectional	6321 participants from NHANES 2003–2006 cohort (3230 w/3091 m, 47.9 yr)	7-day measurement period. A valid day consisted of > 10 h of wear time, with a minimum of 4 valid days	Actigraph 7164 (uniaxial accelerometer, hip/waist-worn, 60 s)	F: not studied I: MPA 2020–5998 cpm/VPA ≥ 5999 cpm/MVPA ≥ 2020 cpm (categorically calculated with cut-points) T: ≥ 1 min / ≥ 10 min (with drop time: “with allowance for interruptions of 1 or 2 min below the cut point”) T: daily physical activity	Troiano et al., [108]
PAGAC	Loprinzi, [50]	Cross-sectional	4584 participants from NHANES 2003–2006 cohort (2269 w/2315 m, 44.6 yr)	7-day measurement period. A valid day consisted of > 10 h of wear time, with a minimum of 4 valid days	Actigraph 7164 (uniaxial accelerometer, hip/waist-worn, 60 s)	F: not studied I: MVPA ≥ 2020 cpm (categorically calculated with cut-point) T: 1–9 min / ≥ 10 min T: daily physical activity	Troiano et al., [108]
PAGAC	Saint-Maurice et al., [67]	Prospective	4840 participants from NHANES 2003–2006 cohort (2580 w/2260 m)	7-day measurement period. A valid day consisted of > 10 h of wear time, with a minimum of 1 valid day	Actigraph 7164 (uniaxial accelerometer, hip/waist-worn, 60 s)	F: not studied I: MVPA ≥ 760 cpm (categorically calculated with cut-point) T: ≥ 1 min / ≥ 5 min (with drop time: “allowed for 1 min of activity counts < 760 cpm”)/ ≥ 10 min (with drop time: “allowed for 2 min of activity counts < 760 cpm”) T: daily physical activity	Matthews, [104] Crouter et al., [92] Welk et al., [110]
PAGAC	Strath et al., [52]	Cross-sectional	3272 participants from NHANES 2003–2004 cohort (1594 w/1678 m, 47.2 yr)	7-day measurement period. A valid day consisted of > 10 h of wear time, with a minimum of 4 valid days	Actigraph 7164 (uniaxial accelerometer, hip/waist-worn, 60 s)	F: not studied I: MVPA ≥ 760 cpm (categorically calculated with cut-point) T: ≥ 1 min/ ≥ 10 min T: daily physical activity	Matthews, [104]
PAGAC	Vasankari et al. [39]	Cross-sectional	1398 participants from Health 2011 study Finland cohort (802 w/596 m, 53.4 yr)	7-day measurement period. A valid day consisted of > 10 h of wear time, with a minimum of 4 valid days	Hookie AM 20 (triaxial accelerometer, hip/waist-worn, 6 s)	F: not studied I: LIPA 1.5–2.9METs/MPA 3–5.9METs/VPA ≥ 6METs/MVPA ≥ 3METs/PA ≥ 1.5METs (calculated with one-minute moving exponential average of the estimated MET values determined from mean amplitude deviation values) T: 30 s–5 min/ ≤ 10 min/ ≤ 15 min/ ≤ 30 min/ > 5 min/ > 10 min / > 15 min/ > 30 min T: daily physical activity	Vaha-Ypya et al., [109]
PAGAC	White et al., [68]	Prospective	2076 participants from CARDIA cohort (1190 w/886 m, 45.2 yr)	7-day measurement period. A valid day consisted of > 10 h of wear time, with a minimum of 4 valid days	Actigraph 7164 (uniaxial accelerometer, hip/waist-worn, 60 s)	F: not studied I: MVPA ≥ 1952 cpm (categorically calculated with cut-point) T: 1–9 min / ≥ 10 min (with drop time: “with allowance for one- or two-minute interruptions below the 1952 count threshold”) T: daily physical activity	Freedson et al., [94]
PAGAC	Wolff-Hugues et al., [40]	Cross-sectional	5668 participants from NHANES 2003–2006 cohort (2868 w/2800 m, 46.5 yr)	7-day measurement period. A valid day consisted of > 10 h of wear time, with a minimum of 4 valid days	Actigraph 7164 (uniaxial accelerometer, hip/waist-worn, 60 s)	F: not studied I: MVPA ≥ 2020 cpm (categorically calculated with cut-point) T: ≥ 10 min (with drop time: “allowing for 1 to 2 min below the 2020 cpm threshold”) T: daily physical activity	Troiano et al., [108]
New search	Ahmadi et al., [69]	Prospective	25,241 participants from UK Biobank study cohort (14178 w/11063 m, 61.8 yr)	7-day measurement period. A valid day consisted of > 16 h of wear time, with a minimum of 3 valid days (at least 1 weekend day)	Axivity AX3 (triaxial accelerometer, dominant-wrist-worn, 10 s)	F: not studied I: two-level PA classification scheme: 1. walking and running/high energetic activities classified with an accelerometer-based activity machine learning classifier; 2. MVPA walking activities ≥ 100 mg and MVPA = running/high energetic activities T: 10 s–1 min/1–3 min/3–5 min/5–10 min T: daily walking activity/daily running-high energetic activity	Pavey et al., [106] Hildebrand et al., [97]
New search	Barone Gibbs et al., [70] (from Brady et al., 126)	Prospective	886 participants from CARDIA cohort (552 w/334 m, 45.2yrs)	7-day measurement period. A valid day consisted of > 10 h of wear time, with a minimum of 4 valid days	Actigraph 7164 (uniaxial accelerometer, hip/waist-worn, 60 s) and Actigraph GT3x (triaxial accelerometer, hip/waist-worn, 60 s)	F: not studied I: MVPA ≥ 1952 cpm (vertical axis either directly from 7164 or reintegrated from GT3x, categorically calculated with cut-point) T: 1–9 min / ≥ 10 min (with drop time: “with allowance for 2 min < 1952 cpm”) T: daily physical activity	Freedson et al., [94]
New search	Cassidy et al., [53]	Cross-sectional	52,556 participants from UK Biobank cohort (27544 w/24880 m)	7-day measurement period, with a minimum of 3 wearing days	Axivity AX3 (triaxial accelerometer, wrist-worn, NK)	F: not studied I: MVPA ≥ 100 mg (categorically calculated with cut-point) T: 1–5 min/ ≥ 10 min T: daily physical activity	Hildebrand et al., [97]
New search	Chen et al., [55]	Cross-sectional	1740 participants from Hisayama study cohort (698 w/1042 m)	7-day measurement period. A valid day consisted of > 10 h of wear time, with a minimum of 4 valid days	Active style Pro HJA-350IT (triaxial accelerometer, hip/waist-worn, 60 s)	F: not studied I: MPA 3–5.9METs/MVPA ≥ 3METs/VPA ≥ 6METs (monitor calculated) T: ≥ 1 min/ ≥ 10 min (with drop time: “with an allowance for up to 2 min below threshold”/only MVPA and VPA ≥ 10 min bouts) T: daily physical activity	Ohkawara et al., [105]
New search	Chen et al., [54]	Cross-sectional	819 participants from Itoshima Frail study cohort (424 w/395 m, 70.9 yr)	7-day measurement period. A valid day consisted of > 10 h of wear time, with a minimum of 4 valid days	Active style Pro HJA-350IT (triaxial accelerometer, hip/waist-worn, 60 s)	F: not studied I: MVPA ≥ 3METs (monitor calculated) T: 1–9 min/ ≥ 10 min (with drop time: “with an allowance for up to 2 min out of 10 to drop below the MVPA intensity threshold”) T: daily physical activity	Ohkawara et al., [105]
New search	De Winter et al., [56]	Cross-sectional	2446 obese participants from NHANES 2003–2006 cohort	7-day measurement period. A valid day consisted of > 10 h of wear time, with a minimum of 4 valid days	Actigraph 7164 (uniaxial accelerometer, hip/waist-worn, 60 s)	F: not studied I: MVPA ≥ NK (categorically calculated with age-specific cut-points) T: ≥ 1 min/ ≥ 5 min / ≥ 10 min/ ≥ 30 min / ≥ 60 min T: daily physical activity	Troiano et al., [108]
New search	Debache et al., [57]	Cross-sectional	131 participants from RECORD study cohort (84 w/47 m, 50.5 yr)	7-day measurement period. A valid day consisted of > 10 h of wear time, with a minimum of 4 valid days	Vitamove Research-V1000^®^ (triaxial accelerometer, chest-worn and thigh-worn, NK)	F: not studied I: MVPA (monitor calculated) T: ≥ 1 min (“the threshold for the proportion of the behavior of interest was kept at 0.8”) T: daily physical activity	Unknown: no validity study is cited
New search	Del Din et al., [58]	Cross-sectional	65 participants (41 w/24 m,73.9yrs)	7-day measurement period, valid for a minimum of 3 full days	Axivity AX3 (triaxial accelerometer, lower-back-worn, NK)	F: not studied I: not studied T: ≥ 1 min T: daily walking activity	Hickey et al., [96]
New search	Diaz et al., [71]	Prospective	7999 participants from REGARDS study cohort (3672 w/4327 m, 63.5 yr)	7-day measurement period. A valid day consisted of > 10 h of wear time, with a minimum of 4 valid days	Actical (omnidirectional accelerometer, hip/waist-worn, 60 s)	F: not studied I: LIPA 50–1064 cpm/MVPA ≥ 1065 cpm (categorically calculated with cut-points) T: ≥ 1 min T: daily physical activity	Hooker et al., [98]
New search	Dos Santos et al., [59]	Cross-sectional	425 participants from EpiFloripa Ageing study cohort (265 w/160 m, 71.8 yr)	7-day measurement period. A valid day consisted of > 10 h of wear time, with a minimum of 4 valid days	Actigraph GT3X and GT3X + (NK, hip/waist-worn, 60 s)	F: not studied I: MVPA ≥ 1952 cpm/MVPA ≥ 1040 cpm/MVPA ≥ 2020 cpm (categorically calculated with cut-points) T: ≥ 1 min/ ≥ 10 min T: daily physical activity	Copeland and Esliger, [90] Troiano et al., [108] Freedson et al., [94]
New search	Jackson et al., [60]	Cross-sectional	375 participants enrolled in a behavioral weight-loss program (298 w/77 m, 45.2yrs)	7-day measurement period. A valid day consisted of > 10 h of wear time, with a minimum of 4 valid days	SenseWear^®^ Pro3 Armband (multisensor device with triaxial accelerometer, upper arm-worn, 60 s)	F: not studied I: MVPA ≥ 3METs (monitor calculated) T: ≥ 1 min/ ≥ 10 min/ < 10 min T: daily physical activity	St-Onge et al., [107] Jakicic et al., [99]
New search	Jefferis et al., 2019a	Prospective	1274 participants from British Regional Heart study cohort (1274 m, 78.4 yr)	7-day measurement period. A valid day consisted of > 10 h of wear time, with a minimum of 3 valid days	Actigraph GT3x (uniaxial accelerometer, hip/waist-worn, 60 s)	F: not studied I: LIPA 100–1040 cpm/MVPA > 1040 cpm (categorically calculated with cut-points) T: 1–9 min/ ≥ 10 min T: daily physical activity	Copeland and Esliger, [90]
New search	Jefferis et al., 2019b	Prospective	1274 participants from British Regional Heart study cohort (1274 m,78.4 y)	7-day measurement period. A valid day consisted of > 10 h of wear time, with a minimum of 3 valid days	Actigraph GT3x (uniaxial accelerometer, hip/waist-worn, 60 s)	F: not studied I: LIPA 100–1040 cpm/MVPA>1040 cpm (categorically calculated with cut-points) T: ≥ 1 min/1–9 min / ≥ 10 min T: daily physical activity	Copeland and Esliger, [90]
New search	Kehler et al. [65] (from Brady et al., 126)	Cross-sectional	2317 participants from NHANES 2003–2006 cohort (1143 w/1174 m, 67.4 yr)	7-day measurement period. A valid day consisted of > 10 h of wear time, with a minimum of 4 valid days	Actigraph 7164 (uniaxial accelerometer, hip/waist-worn, 60 s)	F: not studied I: MVPA ≥ 2020 cpm (categorically calculated with cut-point) T: 1–9 min/ ≥ 10 min (with drop time: “with two allowable consecutive minutes out of 10 min to drop below the MVPA intensity threshold”) T: daily physical activity	Troiano et al., [108]
New search	Lindsay et al., [61]	Cross-sectional	12,002 participants from Fenland study cohort (6428 w/5574 m)	6-day measurement period, valid for a minimum of 72 wearing hours	Actiheart (multisensor device with uniaxial accelerometer, chest-worn, 60 s)	F: not studied I: MVPA ≥ 3METs (from heart rate combined with acceleration in a branched equation model) T: ≥ 1 min/ ≥ 10 min T: daily physical activity	Brage et al., [87]
New search	Millard et al., [74]	Prospective	79,503 participants from UK Biobank study cohort (43307 w/36196 m, 55.9yrs)	7-day measurement period, valid for a minimum of 72 wearing hours	Axivity AX3 (triaxial accelerometer, dominant-wrist-worn, 60 s)	F: not studied I: MVPA ≥ 100 mg (categorically calculated with cut-point) T: 1-15 min/16–40 min / ≥ 41 min/1–9 min/ ≥ 10 min T: daily physical activity	Hildebrand et al., [97]
New search	Mitchell et al., [62]	Cross-sectional	168 participants (132 w/36 m, 50.7 yr)	7-day measurement period. A valid day consisted of > 10 h of wear time, with a minimum of 4 valid days	GENEActiv (triaxial accelerometer, non-dominant wrist-worn, 60 s)	F: not studied I: LIPA 377–805 g.min/MVPA < 806 g.min (categorically calculated with adjusted cut-point for the 100 Hz sampling frequency) T: ≥ 1 min / ≥ 10 min* T: daily physical activity * only MVPA	Esliger et al., [93]
New search	Ostendorf et al., [85]	Case–control study	Participants from the National Weight Control Registry and the Denver metropolitan area	7-day measurement period. A valid day consisted of > 10 h of wear time, with a minimum of 4 valid days (at least 1 weekend day)	activPAL (triaxial accelerometer, thigh-worn, 1 s)	F: not studied I: MVPA stepping event ≥ 75steps/min (monitor calculated) T: ≥ 1 s/ ≥ 10 min T: daily walking activity	Lyden et al., [101]
New search	Roe et al., [75]	Prospective	2816 participants from Osteoporotic Fractures in Men Study cohort (2816 m, 79.1yrs)	7-day measurement period. A valid day consisted of > 90% of wear time over 24 h, with a minimum of 5 valid days	SenseWear® Pro3 Armband (multisensor device with triaxial accelerometer, upper arm-worn, 60 s)	F: not studied I: ≥ 1.5 METs (monitor calculated) T: ≥ 5 min T: daily physical activity	Mackey et al., [103]
New search	Ryan et al., [63]	Cross-sectional	93 participants (51w/42 m, 73.8 yr)	7-day measurement period. Valid for a minimum of 6 wearing days	GENEActiv (triaxial accelerometer, dominant thigh-worn, 10 s)	F: not studied I: MVPA ≥ 3 METs (calculated with an in-house developed data analysis software) T: 10 s–9 min50 s / ≥ 10 min T: daily physical activity	Wullems et al., [111]
New search	Sabag et al., [76]	Prospective	29,836 participants from the UK Biobank study cohort (15862 w/13974 m, 62.2yrs)	7-day measurement period. A valid day consisted of > 16 h of wear time, with a minimum of 3 valid days	Axivity AX3 (triaxial accelerometer, dominant-wrist-worn, 60 s)	F: studied using restricted cubic splines with knots at the 10th, 50th, and 90th percentiles, with the reference group set to zero bouts per day I: two-level PA classification scheme: 1. walking and running/high energetic activities classified with an accelerometer-based activity machine learning classifier; 2. MVPA walking activity ≥ 100 mg and MVPA = running/high energetic activities T: ≥ 3 min T: daily walking activity/daily running-high energetic activity	Pavey et al., [106] Hildebrand et al., [97]
New search	Shiroma et al., [77]	Prospective	3438 participants from NHANES 2003–2006 cohort (1897 w/1541 m, 57.1yrs)	7-day measurement period. A valid day consisted of > 10 h of wear time, with a minimum of 6 valid days	Actigraph 7164 (uniaxial accelerometer, hip/waist-worn, 60 s)	F: not studied I: MVPA ≥ 1952 cpm (categorically calculated with cut-point) T: ≥ 1 min / ≥ 10 min (with drop time: “with 1- or 2-min allowance below the MVPA threshold”) T: daily physical activity	Freedson et al., [94]
New search	Stamatakis et al., [78]	Prospective	25,241 participants from UK Biobank study cohort (14185 w/11056 m, 61.8 yr)	7-day measurement period. A valid day consisted of > 16 h of wear time, with a minimum of 3 valid days (at least 1 weekend day)	Axivity AX3 (triaxial accelerometer, dominant-wrist-worn, 10 s)	F: not studied I: two-level PA classification scheme: 1. walking and running/high energetic activities classified with an accelerometer-based activity machine learning classifier; 2. VPA walking activities ≥ 400 mg and VPA = running/high energetic activities T: 10 s–1 min/10 s–2 min T: daily walking activity/daily running-high energetic activity	Pavey et al., [106] Hildebrand et al., [97]
New search	Stamatakis et al., [79]	Prospective	22,398 participants from UK Biobank study cohort (12276 w/10122 m, 62 yr)	7-day measurement period. A valid day consisted of > 16 h of wear time, with a minimum of 3 valid days (at least 1 weekend day)	Axivity AX3 (triaxial accelerometer, dominant-wrist-worn, 10 s)	F: not studied I: two-level PA classification scheme: 1. walking and running/high energetic activities classified with an accelerometer-based activity machine learning classifier; 2. VPA walking activities ≥ 400 mg and VPA = running/high energetic activities T: 10 s–1 min/10 s–2 min T: daily walking activity/daily running-high energetic activity	Pavey et al., [106] Hildebrand et al., [97]
New search	Verswijveren et al., [64]	Cross-sectional	221 participants from Mitchelstown rescreen study cohort (108 w/113 m, 65.1 yr)	7-day measurement period. A valid day consisted of > 10 h of wear time, with a minimum of 4 valid days	activPAL3 Micro (triaxial accelerometer, thigh-worn, NK)	F: not studied I: stepping (monitor and software classified) T: ≥ 10 min T: daily walking activity	Unknown: no validity study is cited
New search	Wanigatunga et al., [80]	Prospective	548 participants from Baltimore Longitudinal study of Aging cohort (262 w/286 m, 75.8 yr)	7-day measurement period. A valid day consisted of < 5% of missing data, with a minimum of 3 valid days	Actiheart (multisensor device with uniaxial accelerometer, chest-worn, 60 s)	F: not studied I: active ≥ 10 cpm (categorically calculated with cut-point) T: 1–4 min/5–9 min / ≥ 10 min T: daily physical activity	Unknown: no validity study is cited
New search (manually added)	Ahmadi et al., [84]	Prospective	71,893 participants from UK Biobank study cohort (40189 w/31704 m, 62.5 yr)	7-day measurement period. A valid day consisted of > 16 h of wear time, with a minimum of 4 valid days (at least 1 weekend day)	Axivity AX3 (triaxial accelerometer, dominant-wrist-worn, 10 s)	F: not studied I: two-level PA classification scheme: 1. walking and running/high energetic activities classified with an accelerometer-based activity machine learning classifier; 2. VPA walking activities ≥ 400 mg and VPA = running/high energetic activities T: 10 s–2 min T: daily walking activity/daily running-high energetic activity	Pavey et al., [106] Hildebrand et al., [97]
New search (manually added)	Chen et al., [82]	Prospective	3991 participants from the UK-based Whitehall II accelerometer sub-study cohort (1030 w/2961 m, 69.4yrs)	9-day measurement period. A valid day consisted of > 10 h of wear time, with a minimum of 4 valid days	GENEActiv (triaxial accelerometer, non-dominant wrist-worn, 60 s)	F: not studied I: LIPA 40–99 mg/MVPA ≥ 100 mg T: 1–9 min / ≥ 10 min T: daily physical activity	Hildebrand et al., [97]
New search (manually added)	Ekelund et al., [86]	systematic review and harmonized meta-analysis	36,383 participants from 8 cohort studies -WAT2D, REGARDS, ABC, BRHS, WHS, FHS, NHANES, NNPAS- (26487 w/9896 m, 62.6 yr)	7-day measurement period. A valid day consisted of > 10 h of wear time, with a minimum of 4 valid days	Multiple (uniaxial accelerometers, multiple, 60 s)	F: not studied I: Actigraph—LIPA 101–1951 cpm/low-LIPA 101–759 cpm/high-LIPA 760–1951 cpm/VPA ≥ 5725 cpm/MVPA ≥ 1952 cpm. Actical—LIPA 101–1534 cpm/low-LIPA 101–599 cpm/high-LIPA 600–1534 cpm/VPA ≥ 3960 cpm/MVPA ≥ 1535 cpm (categorically calculated with cut-points) T: ≥ 1 min / ≥ 10 min (with drop time: “allowing for 1–2 min drops below the threshold during each period of 10 or more minutes”/only MVPA ≥ 10 min bouts) T: daily physical activity	Colley and Tremblay, [89] Freedson et al., [94]
New search (manually added)	Stamatakis et al., [83]	Prospective	22,368 participants from UK Biobank study cohort (13018 w/9350 m, 61.9yrs)	7-day measurement period. A valid day consisted of > 16 h of wear time, with a minimum of 3 valid days (at least 1 weekend day)	Axivity AX3 (triaxial accelerometer, dominant-wrist-worn, 10 s)	F: not studied I: two-level PA classification scheme: 1. walking and running/high energetic activities classified with an accelerometer-based activity machine learning classifier; 2. VPA walking activities ≥ 400 mg and VPA = running/high energetic activities T: 10 s–1 min/10 s–2 min T: daily walking activities/daily running-high energetic activities	Pavey et al., [106] Hildebrand et al., [97]
New search (manually added)	Yerramalla et al., [81]	Prospective	3991 participants from the UK-based Whitehall II accelerometer sub-study cohort (1030 w/2961 m, 69.4yrs)	9-day measurement period. A valid day consisted of ≥ 2/3 of wear time during the waking period, with a minimum of 4 valid days (at least 2 weekend days)	GENEActiv (triaxial accelerometer, non-dominant wrist-worn, 60 s)	F: not studied I: LIPA 40–99 mg/MVPA ≥ 100 mg T: ≥ 1 min T: daily physical activity	Hildebrand et al., [97]

Drop time, a tolerance—or allowable duration below a given PA threshold—that can interrupt an activity bout, thereby extending the ending of the current activity bout; Epoch length, a predefined fixed time interval over which sensor signals are aggregated to compute a given activity metric

*FITT* Frequency Intensity Time Type, *LIPA* light-intensity physical activity, *m* men, *MPA* moderate physical activity, **MVPA** moderate-to-vigorous physical activity, *NK* not known, *VPA* vigorous physical activity, *w* women, *yrs* years

^a^Precise duration of the epoch is not known

Second, for each of the validation studies that were retrieved (Part B), the framework proposed by the INTERLIVE network was used to extract specific information related to the determination of the validity of consumer wearable activity monitors [13, 14]. The information extracted was related to the following domains (D) of analysis: (D1) target population, (D2) criterion measure, (D3) index measure, (D4) testing conditions, (D5) processing and (D6) statistical analysis. The processing domain D5 was adapted to include the bout definition key variable, which was reported on the basis of the FITT principle [9]. Finally, the main results for performance in bout detection were reported for each study.

### Study Risk-of-Bias Assessment

According to the aim of the present systematic review, risk-of-bias assessment was only conducted on validation studies from which the accuracy of PA bout detection was directly or indirectly addressed (Part B). The risk of bias in suitable validation studies was assessed via a modified version of the Quality Assessment of Diagnostic Accuracy Studies (QUADAS-2) tool [15]. This tool comprises four domains: patient selection, index test, reference standard, and flow and timing. Following the QUADAS-2 guidelines, we selected a set of signaling questions for each domain and added modified questions from the QUADAS-2 background document on the basis of recommendations and expert statements for validation studies [13, 14, 16] and considering the specific aim of PA bout detection (supplemental material 4). For validation studies with multiple experimental steps (e.g., laboratory and field experiments), the risk-of-bias assessment was examined separately for each experimental step.

The risk-of-bias assessment was independently conducted by two authors (AC and ALF). Any discrepancies were resolved through discussion until a consensus was reached. Study quality was evaluated at the domain level: if all signaling questions within a domain were answered with “yes”, the risk of bias was classified as “low”. If any signaling question was answered with “no”, the risk was considered “high”. The “unclear” category was applied only when insufficient data were available for evaluation. On the basis of domain-level ratings, we employed the decision tree proposed by Giurgiu et al. [17] to assess overall study quality, categorizing it as “low risk”, “some concerns”, or “high risk”.

### Synthesis Methods

#### Data Tabulation

The extracted data were synthesized in two separate tables, one presenting the items related to epidemiological studies and another presenting the items related to validation studies.

#### Statistical Analysis and Data Visualization

The methodological features of the included epidemiological studies (Part A)—device model, device position, epoch length, use of a “drop time” (grace period; see Table 1 legend for definition), bout duration, and start year of study data collection—were analyzed using R software (version 4.2.2) to determine their interdependency. For studies that used multiple methodologies (e.g., different bout durations), each methodology was considered separately. Fisher’s exact test with Monte Carlo simulation was used to assess the dependencies between categorical variables. A significance threshold of *p* < *0.05* was applied, and *p* values were adjusted for multiple comparisons using the Benjamini–Hochberg procedure [18]. Effect sizes were calculated as Cramér’s V and interpreted using Cohen’s thresholds: negligible (< 0.1), small (0.1–0.29), medium (0.3–0.49), large (0.5–0.69), very large (0.7–0.89), and extremely large (≥ 0.9) [19]. A Sankey diagram (*ggalluvial* [20]) and a hierarchical tree diagram (*ggtree* [21]) were generated for data visualization.

#### Reporting Bias Assessment

The review of the authors’ judgments about each domain of the risk-of-bias item and for each suitable validation study (Part B) was reported graphically via the robvis tool [22]. The proportion of studies with each level of bias (‘low risk’, ‘high risk’, and ‘unclear risk’) for each bias domain and for overall studies was also reported graphically.

## Results

### Part A: Epidemiological Studies About PA Bout Duration and Health Outcomes

#### Search Results

**Fig. 1:**
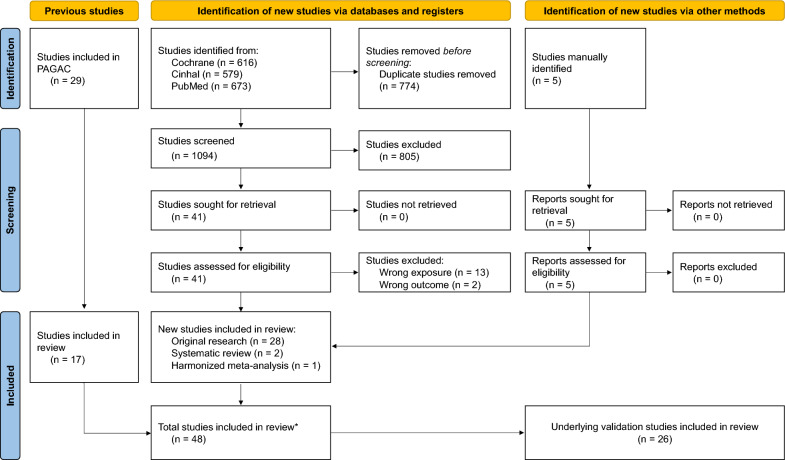
Preferred reporting items for systematic reviews and meta-analyses flow diagram for the search and selection process. ^*^ As mentioned in the methodology section, the two systematic reviews were not counted in the total number of studies, but two new original studies identified from these systematic reviews were added to this total

Figure 1 displays the flowchart of the search and selection process. Among the 46 studies sought for retrieval and then assessed for eligibility, 15 studies were excluded [24–38]. The main reasons were wrong exposure (e.g., overall PA but not PA bouts studied) and/or wrong outcome (e.g., health outcome not included in the inclusion criteria). A total of 48 epidemiological studies were finally included: 27 cross-sectional studies [39–65], 19 prospective studies [66–84], one case‒control study [85] and one harmonized meta-analysis [86]. Seventeen studies [39–52, 66–68] originated from the PAGAC update by Jakicic et al. [12], and 31 studies [53–65, 69–86] originated from the new search.

#### Characteristics of Epidemiological Studies

Table 1 describes the main characteristics of the included epidemiological studies underlying the devices and methods used for PA bout determination. Analysis of device type, position, epoch length, drop time, bout duration, and start year of study data collection across the 48 included epidemiological studies revealed 113 methodological instances, representing 68 unique combinations, underscoring the diversity in practices.

**Fig. 2:**
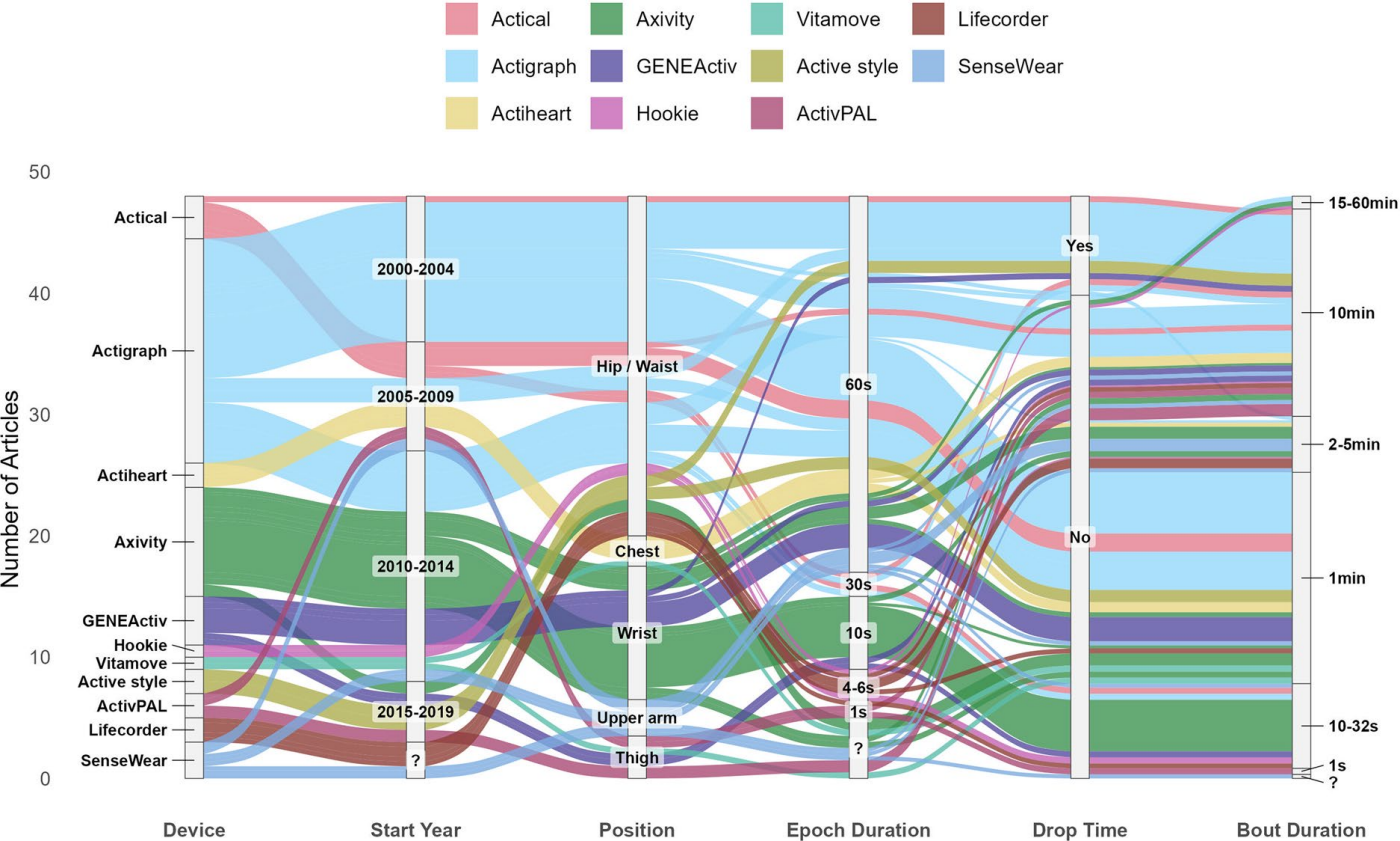
Sankey diagram of the epidemiological study characteristics Legend. The Sankey diagram illustrates the methodological combinations across the included epidemiological studies. This diagram depicts the co-occurrence and relative prevalence of methodological choices. Each band represents the flow of studies adopting specific parameter combinations, with the bandwidth proportional to the frequency of studies

Overall, the most commonly used devices in the literature thus far are Actigraph (36% of the combinations) and Axivity (18%). Devices were most often worn on the hip/waist (62%) or the wrist (21%). The most common epoch duration is 60 s (59%), followed by epoch ≤ 10 s (29%). The majority of studies did not use a drop time in their definition of the bout (86%). PA bouts of at least 1 or 10 min were the most common duration studied in the literature (33% each). A Sankey diagram (Fig. 2) illustrates the prevalence of these combinations, whereas a hierarchical tree diagram (supplementary material 5) highlights their chronological evolution and study-specific associations.

#### Analysis of Epidemiological Studies

Strong interdependencies were observed between parameters (supplemental material 6). The device model and position showed the strongest relationship (*p* < 0.001, *V* = 0.93), with Axivity devices predominantly used at the wrist and Actigraph or Actical exclusively at the hip. Device type was also linked to epoch duration (*p* < 0.001, *V* = 0.73). For example, Actigraph studies were more likely to use 60-s epochs, whereas Axivity predominantly used 10-s epochs. Position and epoch duration were similarly associated (*p* < 0.001, *V* = 0.50), with shorter epochs (10 s) more frequently applied at the wrist and longer epochs (60 s) at the hip.

Methodological approaches evolved significantly over time, with the start year of study data collection associated with all the studied factors, such as the device model (*p* < 0.001, *V* = 0.79), position (*p* < 0.001, *V* = 0.46), epoch duration (*p* < 0.001, *V* = 0.46), drop time (*p* = 0.003, *V* = 0.37) and bout duration (*p* = 0.015, *V* = 0.31). Earlier studies (2000–2004) exclusively employed Actigraph or Actical devices at the hip with 60-s epochs. Epoch durations diversified over time, with 30-s epochs emerging in 2005–2009 and 10 s or shorter epochs becoming widespread from 2010–2014. From 2005–2009, device type and placement also diversified, with ActivPAL at the thigh, SenseWear at the upper arm, and Actiheart at the chest.

Bout-related parameters showed additional patterns. Longer epochs were associated with longer bout thresholds (*p* < 0.001, *V* = 0.48) and the presence of drop time (*p* = 0.021, *V* = 0.35). Drop time itself was associated with bout duration (*p* < 0.001, *V* = 0.53), as 93% of the studies employing drop time focused on bouts of at least 10 min. The device model was also associated with bout duration (*p* < 0.001, *V* = 0.41) and drop time (*p *= 0.023, *V* = 0.43), with Axivity often linked to shorter bouts (< 1 min) without drop time and Actigraph or Actical to longer bouts (≥ 1 min) with drop time.

### Part B: Validation Studies About PA Bout Detection

#### Characteristics of the Validation Studies

Among the 48 epidemiological studies reviewed, 26 distinct validation studies were identified [87–112] (supplemental material 7). Three epidemiological studies did not reference any validation study to support their methodology in terms of PA bout detection [57, 64, 80]. Upon further analysis, two of the identified validation studies were not formal validation studies [104, 108]. Indeed, those studies by Matthews et al. [104] and Troiano et al. [108] only proposed new PA intensity thresholds on the basis of previous publications but not on a validation protocol. Taken together, the two most cited validation studies were those by Hildebrand et al. [97] (*n* = 10) and Troiano [108] (*n* = 10), referenced in 20 out of 48 studies, representing approximately 40%. For most validation studies (85%), the accuracy of PA bout duration and occurrence assessment has not been formally studied but mainly focused on the accuracy of PA bout intensity estimation. Consequently, only four of the 26 available validation studies were deemed suitable for addressing bout performance detection [96, 101, 106, 111] (Table 2). These four studies accounted for less than 20% of the selected epidemiological studies.

**Table 2:** Characteristics of validation studies suitable for assessing bout performance detection

Validation study (native epidemiological study)	D1: target population	D2: Criterion measure (type, location, epoch)	D3: Index measure (type, location, epoch)	D4: testing conditions	D5: processing	D5 (continued): bout definition	D6: statistical analysis	Main results for performance in bout detection (index measure validity)
Hickey et al., 2017 [58]	10 healthy participants (27.5 y)	Step counting and bout identification using direct observation (GoPro HERO, chest, NA)	Axivity AX3 (triaxial accelerometer, lower-back-worn, 0.1 s)	*Setting*. Free-living *Protocol*. Two sessions (1 h) of usual activities outside the laboratory (daily-life and locomotive activities including cycling)	*Criterion data*. Step and bout count were performed using ELAN Linguistic Annotator. All events (walking, postural transitions, activities) were recorded with their relative contextual information (location, purpose, duration, etc.). All periods of non-walking activity were removed and step events were collated into their respective bouts with a minimum resting period of 2.5 s between bouts. All bouts less than three steps were removed *Index data*. A two-stage approach algorithm for processing and gait detection was used to detect walking activity *Method*. Method was tested using direct observation comparison *Sync*. Yes (gesture recognition)	F: not defined I: not defined T: ≥ 0.5 s T: daily walking activity	Spearman’s correlations ICC Bland–Altman plots	Bout count: relative—rho = 0.909, *p* < 0.0005 (Spearman correlations)/absolute—ICC(2,1) = 0.941, *p* ≤ 0.0005
Lyden et al., 2017 [85]	13 healthy participants (8 w/5 m, 24.8 y)	Activity classification using direct observation (NA, NA, NA)	ActivPAL (triaxial accelerometer, thigh-worn, 1 s)	*Setting*. Free-living *Protocol*. Three days of usual activities outside the laboratory	*Criterion data*. A handheld personal digital assistant was used to record and time-stamp participant behavior (activity type, intensity, and duration) *Index data*. Activities were directly classified by the device ("event" data file). MET value assigned to standing events was adjusted from 1.4 (default value) to 1.5 METs. Then, to determine total time spent in activity intensity categories, a customized R program to extrapolate events files to a 1 s epoch data file was used *Method*. Method was tested using direct observation comparison *Sync*. NK	F: not defined I: LIPA/MVPA using internal device algorithm based on a cadence-based linear regression T: ≥ 1 s / ≥ 10 min T: daily physical activities	Bias (95% confidence interval) rMSE ICC two-way ANOVA Bland–Altman plots	Agreements between direct observation and all AP estimates (range ICC: 0.78–0.99, P < 0.05) Guideline bouts: Bias = −0.3, rMSE = 0.7, ICC = 0.92 Guideline minutes: Bias = −5.4, rMSE = 17.3, ICC = 0.91 MVPA (min): 68.3: Bias = −2.6, rMSE = 8.4, ICC = 0.98 Light (min): Bias = 1.7, rMSE = 12.3, ICC = 0.99
Pavey et al., 2017 [69, 76, 78, 79, 83, 84]	21 participants (27.6 y)	Activity classification using direct observation (NA, NA, NA)	GENEActiv (triaxial accelerometer, wrist-worn, 10 s)	*Setting*. Semi free-living *Protocol*. Seven semi-structured activities (3 min) in the laboratory (sedentary activities, household activities, locomotive activities)	*Criterion data*. The participant had to conform to the protocol and a research assistant timed the activities *Index data*. Raw acceleration data from the middle 2 min of each activity were parsed and segmented into epoch of 10 s for feature extraction. Random forest model was then trained and applied to determine activity class (sedentary, stationary + , walking and running) *Method*. Classification model was defined using random forest model *Sync*. NK	F: not defined I: not defined T: ≥ 10 s T: daily physical activities	Leave-one-out cross-validation Confusion matrix (sensitivity, specificity, PPV, NPV, and balanced accuracy)	Laboratory trial Walk: Sensitivity = 0.92, Specificity = 0.99, PPV = 0.94, NPV = 0.99, Balanced accuracy = 0.95 Run: Sensitivity = 0.94, Specificity = 0.99, PPV = 0.98, NPV = 0.99, Balanced accuracy = 0.97 Weighted Kappa = 0.88 ± 0.12 Free-living trial Stepping vs. non-stepping: Accuracy = 93.7 (2.4%), Sensitivity = 53.8 (11.5%), Specificity = 96.3 (1.4%), PPV = 47.7 (11.7%), NPV = 96.9 (1.7%), Balanced accuracy = 75.1 (5.7%), Kappa = 0.47 (0.10) Stepping time estimates: ICC = 0.92 (95% CI = 0.75–0.97) Mean bias = −10.3 min/d (95% LOA = −46.0 to 25.4 min/d)
Wullems et al., 2017 [63]	40 older healthy participants (20 w/20 m, 73.5 yrs)	Oxygen uptake estimation using indirect calorimetry (Douglas bag, NA, 60 s) Direct observation (NA, NA, NA)	GENEActiv (triaxial accelerometer, thigh-worn, 10 s)	*Setting*. Semi free-living *Protocol*. Ten semi-structured activities (4 min) in the laboratory (sedentary activities, locomotive activities including cycling)	*Criterion data*. VO2 values over the final 2 min of each bout were considered to represent each activity. Then, values were expressed in METs. Intensity classification for each sample was done by checking the MET value and the participant’s posture using the video recording *Index data*. Raw acceleration data from the final 2 min of each activity were parsed and segmented into epoch of 10 s for feature extraction. Random forest model was then trained and applied to determine activity class (Sedentary/Standing/LIPA/MVPA) *Method*. Methods were tested using indirect calorimetry comparison *Sync*. Yes	F: not defined I: LIPA and MVPA classification according to a random forest model T: ≥ 10 s T: daily physical activities	Leave-one-subject-out method Confusion matrices (sensitivity, specificity, balanced accuracy) Independent T-test One-way ANOVA repeated-measures test	LIPA: Sensitivity = 63.7, Specificity = 97.5, Balanced accuracy = 80.6 MVPA: Sensitivity = 97.3, Specificity = 92.9, Balanced accuracy = 95.1

*ANOVA* analysis of variance, *CI* confidence interval, *ICC* intraclass correlation coefficient, *LIPA* light-intensity physical activity, *m* men, *MVPA* moderate-to-vigorous physical activity, *NA* not-applicable, *NK* not known, *NPV* negative predictive value, *PPV* positive predictive value, *rMSE* root mean squared error, *w* women, *yrs* years

### Risk of Bias in the Validation Studies

**Fig. 3:**
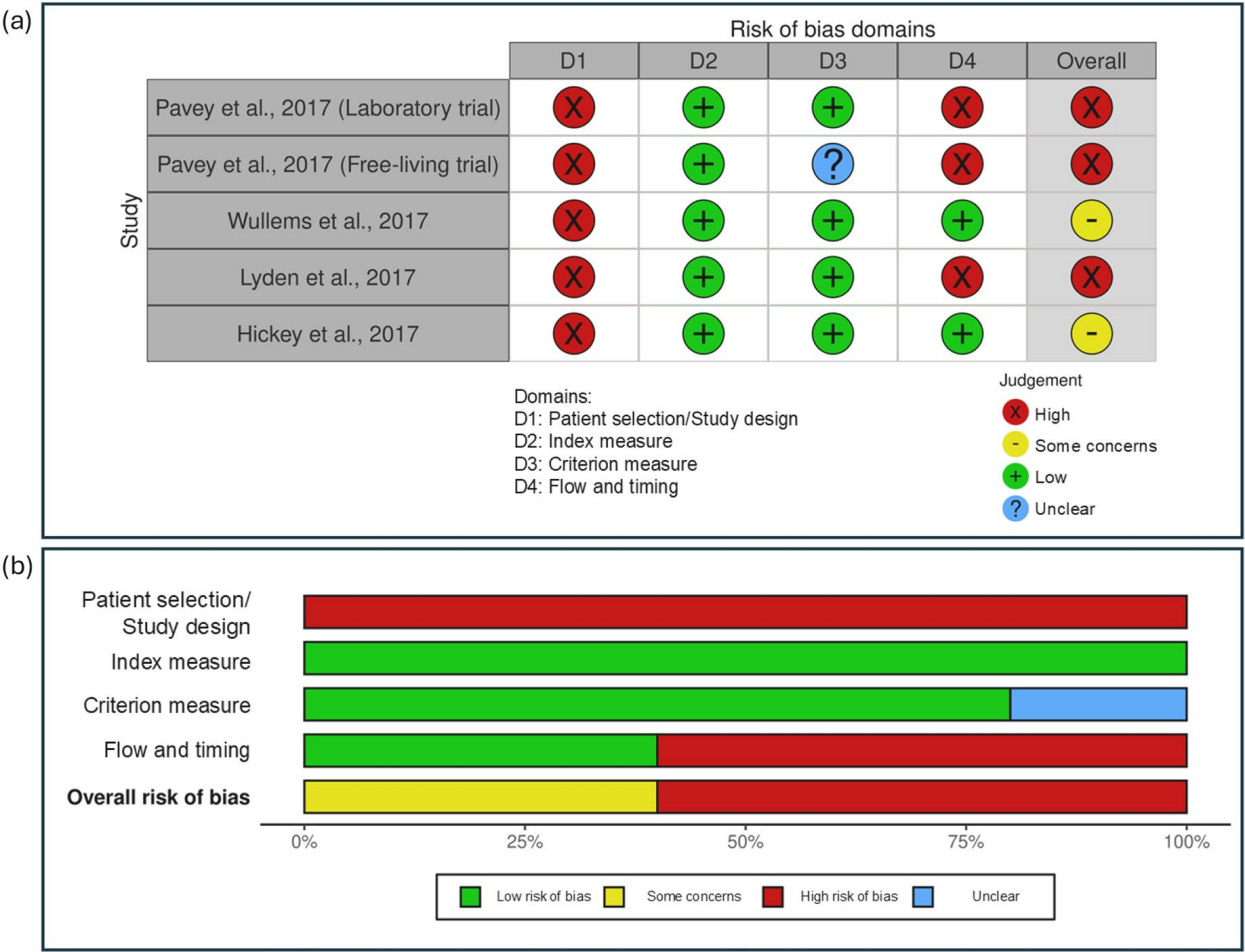
Risk-of-bias assessment via the QUADAS-2 tool Legend. Panel a presents all judgments per study, whereas Panel b summarizes all judgments per item across studies

The review authors’ judgments about each risk of bias for each domain are reported in Fig. 3. The overall study risk of bias was ranked as “High” or with “some concerns” for all studies or trials. The main concern was with domain #1 since all studies were ranked as “High” risk of bias for this domain due to sample size issues. The second concern was with domain #4 due to the absence of information about data synchronization between the index and reference measures in 2 out of 4 studies. Substantial methodological heterogeneity precluded their inclusion in a meta-analysis, so a qualitative analysis of the four studies was performed.

Hickey et al. [96] validated an algorithm for detecting step and walking bout counts from an Axivity AX3 accelerometer worn on the lower back, using video annotation as the gold standard. The algorithm showed excellent agreement for step count (ICC = 0.975) and walking bout count (ICC = 0.941) but tended to overestimate the number of walking bouts (*Z* = −2.074, *p* = 0.037), particularly for short and fragmented bouts. However, confidence intervals for ICC estimates were not reported, preventing inclusion in a pooled analysis.

Lyden et al. [101] evaluated the ActivPAL thigh-worn accelerometer for activity classification in free-living conditions against direct observation, demonstrating high performance (ICC > 0.98) for time spent in sedentary, light and moderate-to-vigorous physical activity. While ActivPAL performed well for bout detection (ICC > 0.92), it was based on MVPA bouts of at least 10 min, excluding shorter or lower intensity walking bouts, limiting comparability with other studies.

Pavey et al. [106] assessed a random forest machine learning model using wrist-worn GENEActiv accelerometers against either direct observation (laboratory trial) or ActivPAL (free-living trial), and the results revealed high agreement under laboratory conditions (sensitivity = 93.5%, specificity = 98.9%) but reduced performance in free-living settings (sensitivity = 53.8%, specificity = 96.3%), where slow-paced or fragmented walking bouts were often misclassified as non-stepping. ICC estimates (ICC = 0.92 in free-living conditions) were reported only for total stepping time rather than for individual walking bouts, further restricting its relevance for meta-analysis.

Finally, Wullems et al. [111] compared a random forest model to traditional cutoff point methods using thigh-mounted accelerometers in older adults, with the machine learning model outperforming cutoff-based methods and achieving superior classification for light-intensity physical activity (LIPA balanced accuracy = 80.6% vs. ≤ 78.9%; MVPA balanced accuracy = 95.1% vs. ≤ 94.5%). However, only sensitivity and specificity values were provided for bout classification, preventing ICC-based comparisons with other studies.

## Discussion

The present systematic review yielded two primary findings. First, epidemiological studies examining the association between PA bout duration and health outcomes in adults have employed a wide variety of accelerometric methods and devices to assess PA bout duration (Part A). Second, among the underlying validation studies cited in epidemiological research to justify the methods used to assess PA bout duration, only 15% (*n* = 4/26) of those validation studies formally evaluated the accuracy of PA bout detection, accounting for less than 20% of the identified epidemiological studies (Part B).

### Accelerometric-Based Methods in Epidemiological Studies: Heterogeneity and Evolution

The wide variety of accelerometric methods and devices used to assess PA bouts in identified epidemiological studies underscores the need for standardization of methods for this specific issue. Beyond considerably limiting comparability across studies, such heterogeneity raises questions about how it may impact the reported associations between PA bout duration and health. The observed clustering of device type, sensor placement, and epoch length reflects how historical conventions and evolving technologies have shaped the accelerometric methods used in epidemiological research on PA bouts and health and, in turn, how this issue has been addressed.

Previous epidemiological studies (e.g., before 2018–2020) typically employed Actigraph and Actical devices at the hip, using 60-s epochs, an intensity cutoff approach and, most of the time, included a drop time condition in the bout definition. Those studies focused mainly on PA bouts of at least 1 or 10 min. As discussed below, none of those studies used accelerometric methods to detect PA bouts that were formally validated for such purpose. These methods were used due to the absence of more appropriate alternatives considering the features of the devices used (e.g., epoch length).

Furthermore, although commonly used in most epidemiological studies conducted before 2018–2020, the definition of drop time remains unclear and inconsistent across studies (see Table 1), with no evidence regarding its impact on the validity of MVPA bout detection or on actual physiological responses. The concept appears to have emerged from practical considerations aimed at facilitating the detection of MVPA bouts ≥ 10 min in the presence of brief interruption(s), in line with previous PA guidelines [3–5]. The drop-time definition most commonly used to detect MVPA bouts ≥ 10 min was that proposed by Troiano et al.: “10-min activity bouts were defined as 10 or more consecutive minutes above the relevant threshold [i.e., the counts threshold indicative of MVPA], with allowance for interruptions of 1 or 2 min below threshold” [108]. For instance, consider the following sequence: 5 min at MVPA–1 min below MVPA–6 min at MVPA–1 min below MVPA–7 min at MVPA. Without applying a drop-time rule, no MVPA bout would be detected; whereas applying the definition proposed by Troiano et al. would result in a single MVPA bout of 20 min. However, it remains unclear whether drop time must be continuous or may be distributed within a detected PA bout. Moreover, no consensus exists regarding whether limits should be imposed on the number of drop-time occurrences or on its application between successive PA bouts.

Although the extent to which PA bout detection methods influence the observed association between PA bout duration and health remains uncertain, it is likely that some effect is present. Previous works clearly demonstrated how accelerometer parameter choices, such as epoch length, intensity cutoff thresholds, drop time use and minimum-bout duration definitions, substantially influence estimates of MVPA [113–116]. Further, it has been reported that ~46% of walking bouts last 20 s or less, and only 0.5% of walking bouts in daily life last more than 10 min, accounting for ~14% of the total daily walking time [7]. Thus, it is unlikely that an epoch length of 60 s with the combination or not of a drop time use accurately reflects such a daily PA pattern. It should be noted that the latest PA guidelines that proposed the paradigm shift regarding PA bout duration only relied on those former epidemiological studies. As previously highlighted, such changes in the guidelines were based on the absence of evidence (to support a minimum bout duration of 10 min) rather than direct evidence of the health-enhancing properties of shorter and intermittent bouts [117], which could be partly explained by the PA bout detection methods used.

In the last 5 to 7 years, an increasing number of epidemiological studies have used wrist or thigh-worn monitors (e.g., Axivity, GENEActiv, ActivPAL), raw acceleration over shorter epochs (10 s), and machine-learning approaches for intensity-based and/or activity-based recognition. These technological and methodological advances have been associated with a shift in epidemiological issues related to the association between PA patterns and health. Indeed, these recent epidemiological studies revealed new associations between health and accelerometry-measured “PA micropatterns”, defined as short bursts of intermittent PA lasting ≤ 3 min, introducing the concept of Vigorous Intermittent Lifestyle Physical Activity (VILPA) [117]. Taken together, these studies highlight the value of using wearables to quantify the granular characteristics of PA bouts (e.g., FITT components) that may influence health over and above the total of cumulated amount of MVPA [69, 76, 78, 81, 84]. Although more recent and less numerous than former epidemiological studies, there is no consensus either regarding what the best methodological package is to detect and characterize PA bouts. This emphasizes the importance of standardized reporting and methodological harmonization.

### Accelerometric-Based Methods in Validation Studies: Qualitative Analysis

Among the underlying validation studies cited in epidemiological research to justify the methods used to assess PA bout duration, only four out of 26 (15%) formally evaluated the accuracy of PA bout occurrence and duration assessment. The remaining underlying validation studies cited in epidemiological studies were designed primarily to assess the accuracy of PA intensity estimation rather than the accuracy of bout detection. In these studies, consistent with the requirements of gold standard gas exchange measurements to reach a steady state of oxygen uptake to accurately reflect PA intensity, predetermined, standardized, and isolated bouts of PA at constant load and lasting ≥4–5 min were primarily used. Furthermore, most of these validation studies on PA intensity were conducted when accelerometers were limited to 1-min epochs, with the primary goal of determining whether individuals reached sufficient MVPA levels through bouts of 8–10 min. This made finer-resolution measurements less relevant but necessitated aggregation rules for combining discrete 1-min PA bouts, as reflected by the drop-time issue discussed above, to accurately identify longer bouts. Importantly, this discrepancy between the PA bout detection methods used in epidemiological studies and those used in the underlying validation studies does not necessarily imply inaccuracy. The issue does not pertain to the quality of the validation studies themselves but rather to the appropriateness of the validated methods for the specific approaches used in epidemiological research.

Collectively, the four remaining identified validation studies exhibited substantial heterogeneity in device type, reference method, and statistical reporting, making direct comparisons difficult and precluding quantitative synthesis through a meta-analysis conducted in accordance with the Cochrane guidelines [23]. Thus, only a qualitative analysis was performed. Following this qualitative assessment, the high risk of bias ranked for the “Patient selection/Study design” domain was mostly due to an insufficient sample size issue, whereas no risk of bias was identified for the other criteria for most studies. Importantly, three out of four studies included a free-living trial in their experimental design, and data collection under true naturalistic conditions is a key methodological point to consider for validation studies of PA monitors [13, 14, 16]. As shown, those studies reported high performance in PA bout detection when algorithms and devices were tested under laboratory conditions and/or when considering cumulative metrics for statistical analysis (e.g., total stepping time or bouts). However, performance decreased significantly in free-living conditions and/or when considering short and fragmented bouts of PA, which directly reflects daily PA patterns [7]. Furthermore, performance in PA bout detection in these studies was primarily assessed at the epoch level. However, momentary accuracy at this level does not necessarily translate into an accurate characterization of discrete walking bouts, i.e., at the bout level [118, 119]. PA patterns often involve a complex alternation of activity and non-activity bouts, many of short duration. Consequently, even minor misclassifications at the epoch level can artificially fragment continuous bouts or obscure the true structure of activity fragmentation, thereby complicating the accurate interpretation of the relationship between PA bout characteristics and health.

The choice of an appropriate gold standard and its alignment with the metric being validated is another crucial factor in the design of studies assessing the validity of PA monitors [16]. A single gold standard may be insufficient to evaluate all dimensions of PA bouts, and different a priori accepted gold standard to assess a given dimension cannot capture strictly the same granularity. The four validation studies identified used three different gold standard methods to assess bout detection: video recording of steps or activities [96, 111], direct observation of participants activities [101, 106], or the activPAL device [106]. It is difficult to quantify the impact of the reference method used on bout detection performance and on the studies comparability, but this likely impacts the granularity of PA quantification that can be achieved. Furthermore, this could partly explain the lack of information about data synchronization between the index and reference measures in the two validation studies [101, 106].

Finally, it is important to emphasize that although the present review focused on the validity of accelerometer devices and methods for detecting and assessing PA bouts of any duration, accurate detection of a PA bout depends not only on its duration but is also influenced by its other components (intensity, type, and frequency), with bout duration and frequency being inherently interrelated.

### Future Directions

At present, it cannot be assumed that available monitors and methods are sufficiently validated to detect PA bouts without considerable risk of bias in free-living conditions. Since the validation evidence presented here was directly related to, and therefore somewhat limited by, the methods used in the retrieved epidemiological studies, it could be argued that more appropriate and valid methods may exist for detecting PA bouts and assessing their components, which would warrant dedicated future systematic review. Although this specific topic deserves further attention, recent and concerning evidence has highlighted the lack of methodologically sound validation studies of wearables for capturing 24 h physical behaviors [120, 121]. Such methodological uncertainty may partly explain the current difficulty in establishing consistent associations between the duration and characteristics of PA bouts and health outcomes. To address these limitations, future research should prioritize the development and validation of PA monitors and measurement methods that can reliably capture PA bouts in real-world settings without introducing systematic bias, while leveraging the most recent technologies to enable fine-grained detection.

From this perspective, the absence of consensus on an ‘optimal’ epoch length—illustrated by the variability in durations used in epidemiological studies (ranging from 4 to 60 s)—underscores the need for approaches that align with human PA patterns in future research. Longer epochs restrict the ability of monitors to detect brief events (e.g., short PA bouts or interruptions). Therefore, valid characterization of activity patterns requires an epoch length consistent with the behaviors being measured. Given the highly fragmented nature of human activity [7], future studies should determine whether shorter epoch lengths are preferable to the conventional 60-s epoch length for better capturing PA fragmentation, as applied in recent epidemiological studies.

Future research should also critically examine the use of drop time, as this ‘grace period’ has, to our knowledge, never been formally validated in any study. Orendurff et al. reported that 82% of pauses between walking bouts last less than two minutes. Consequently, commonly applied drop time criteria inherently exclude these short interruptions, which are nonetheless intrinsic to the natural pattern of human activity. Beyond this behavioral inconsistency with real-life PA patterns, it is important that any drop time criteria align with actual physiological responses. Oxygen uptake remains elevated above resting levels (Excess Post-Exercise Oxygen Consumption, EPOC) for a period after cessation of PA, depending on intensity and duration [122]. This suggests that appropriate drop time criteria might integrate multiple discrete efforts occurring in close succession into a single activity bout, provided that oxygen uptake or EPOC remains at a sufficient level during the grace period [123].

Finally, future validation studies should include free-living assessments using appropriate gold standards. The use of an inappropriate gold standard in available validation studies conducted in free-living contexts has been identified as a major methodological issue [120, 121]. However, to date, only a limited number of studies have utilized these gold standards. Such approaches are essential for evaluating PA bout detection at the bout level, ensuring that cumulative or epoch-level statistical measures accurately reflect bout-level metrics and permit a valid assessment of activity fragmentation.

Taken together, given evidence that physical activity patterns—including the frequency of PA bouts [69, 78, 79]—influence health outcomes, future research should prioritize methods designed for pattern detection rather than approaches relying solely on accumulated PA derived from epoch classification.

## Limitations

No restriction was applied, but participants in the identified epidemiological and validation studies were predominantly from cohorts in high-income countries. The limited access to technologies for monitoring PA in low- and middle-income countries raises concerns about the global understanding of the relationship between device-measured PA and health. Moreover, caution is required when interpreting the findings, as generalizability is not guaranteed for low- and middle-income countries, where differences in socioeconomic conditions, healthcare infrastructures, and lifestyle factors may lead to varying health outcomes. Future research should aim to include more diverse populations to increase the external validity of epidemiological evidence.

## Conclusions

Wearables have the potential to revolutionize PA guidelines and interventions [8]. The incorporation of more scientific evidence based on wearables for the future of PA surveillance and PA guidelines is a key component of the WHO global action plan on PA 2018–2030 [124, 125]. As stated by the WHO, the harmonization of devices and methods is a necessary requirement for wider adoption in national health surveillance systems, result comparability, and global knowledge transfer [124, 125].

This systematic review, by assessing the validity of accelerometer devices and methods in studies underpinning PA guidelines on bout duration and health, underscored the absence of standardized PA bout detection and characterization methods, as well as the lack of strong scientific evidence supporting the employed methodologies.

The present work highlights the need for further research to determine the optimal combination of methodological package (epoch duration, minimum bout, drop time, device, and location) to minimize errors in PA bout detection and characterization. This would in turn help in determining whether varying bout lengths produce distinct effects on health outcomes.

## Supplementary Information

Supplementary Material 1.

Supplementary Material 2.

Supplementary Material 3.

Supplementary Material 4.

Supplementary Material 5.

Supplementary Material 6.

Supplementary Material 7.

## Data Availability

All data generated or analyzed during this study are included in this published article and its supplementary information files.

## Abbreviations

FITT: Frequency, Intensity, Time and Type
MVPA: Moderate to vigorous physical activity
PA: Physical activity
PAGAC: Physical Activity Guidelines Advisory Committee
QUADAS: Quality Assessment of Diagnostic Accuracy Studies
VILPA: Vigorous Intermittent Lifestyle Physical Activity
