# Supplementary material for: Synthesis and Validity of Accelerometer Devices and Methods Used in Epidemiological Studies of Physical Activity Bout Duration and Health Outcomes: A Systematic Review
Source: Sports Med Open. 2026 Jul 1;12:84. doi: 10.1186/s40798-026-01039-4 (PMC13323697; doi:10.1186/s40798-026-01039-4)
Supplement: Supplementary file 2 — Supplementary Material 2. [file 40798_2026_1039_MOESM2_ESM.pdf]

Supplementary file 2: Search Strategy

Search Strategy: PubMed (S1: Original Research)

Database: PubMed; Date of Search: 14/11/2024; 448 results

Set	Search Strategy
Physical activity (bouts)	((“intermittent activity”[tiab] OR “intermittent exercise”[tiab] OR "accumulated activity"[tiab] OR bouts[tiab]) AND ("Physical activity"[tiab] OR "Exercise"[mh] OR "Exercise"[tiab]))
Outcomes (Cardiovascular Disease Incidence OR Mortality OR Cardiometabolic Risk Factors OR Cardiorespiratory Fitness OR Frailty)	AND (((("Arteriosclerosis"[mh] OR "Heart failure"[mh] OR "Myocardial ischemia"[mh] OR "myocardial infarction"[mh] OR "Stroke"[mh] OR "Subarachnoid hemorrhage"[mh] OR "Intracranial hemorrhages"[mh]) OR ((Arterioscler o*[tiab] OR Atheroscler o*[tiab] OR "Cerebral infarction"[tiab] OR "Cerebrovascular diseases"[tiab] OR "Cerebrovascular disease"[tiab] OR "Coronary heart disease"[tiab] OR "Heart failure"[tiab] OR "Intracerebral Hemorrhage"[tiab] OR "Intracerebral Hemorrhages"[tiab] OR "Intracranial hemorrhage"[tiab] OR "Intracranial hemorrhages"[tiab] OR "myocardial infarction"[tiab] OR "Stroke"[tiab] OR "Subarachnoid hemorrhages"[tiab] OR "Subarachnoid hemorrhage"[tiab] OR "Ischemic heart diseases"[tiab] OR "Ischemic heart disease"[tiab]) NOT medline[sb])) AND ("risk"[tiab] OR "risks"[tiab] OR "Incidence"[tiab] OR "incident"[tiab] OR "Death"[mh] OR "Death"[tiab] OR "Dying"[tiab] OR Fatal*[tiab] OR Mortalit*[tiab] OR "Mortality"[mh] OR "Postmortem"[tiab])) OR ("blood pressure"[mh] OR "blood pressure"[tiab] OR "systolic pressure"[tiab] OR "diastolic pressure"[tiab] OR "mean arterial"[tiab] OR "bp response"[tiab] OR "bp decrease"[tiab] OR "bp reduction"[tiab] OR "normotensive”[tiab] OR “hypertension”[tiab] OR “hypotension”[tiab] OR “normotension”[tiab] OR “hypertensive”[tiab] OR “hypotensive”[tiab] OR "Body weight"[mh] OR "Body composition"[mh] OR "Body Mass Index"[mh] OR “Waist circumference”[mh] OR "Body weight"[tiab] OR "Body weight change"[tiab] OR "Weight gain"[tiab] OR "Weight status"[tiab] OR "Overweight"[tiab] OR "Weight Control"[tiab] OR "Weight maintenance"[tiab] OR "Weight regulation"[tiab] OR "Weight stability"[tiab] OR "Body composition"[tiab] OR "Body Mass Index"[tiab] OR “Waist circumference”[tiab] OR "insulin resistance"[mh] OR "glucose intolerance"[mh] OR "glucose control"[tiab] OR "insulin resistance"[tiab] OR "prediabetes"[tiab] OR "pre-diabetes"[tiab] OR "glucose intolerance"[tiab] OR (diabetes[tiab] AND ("type 2"[tiab] OR "type II"[tiab])) OR “cholesterol”[mh] OR “triglycerides”[mh] OR “lipoproteins”[mh] OR “cholesterol”[tiab] OR “triglycerides”[tiab] OR “triglyceride”[tiab] OR “blood lipids”[tiab] OR "lipoprotein"[tiab]) OR (("Cardiorespiratory fitness"[mh] OR "VO2 max"[tiab] OR "maximal oxygen uptake"[tiab] OR "peak oxygen uptake"[tiab] OR "aerobic capacity"[tiab]) OR (("Cardiorespiratory fitness"[tiab]) NOT medline[sb])) OR (((“mortality” [mh] OR “frailty” [mh]) OR (“mortality” [tiab] OR “frailty” [tiab])) AND ("risk"[tiab] OR "risks"[tiab] OR "Incidence"[tiab] OR "incident"[tiab] OR "Death"[mh] OR "Death"[tiab] OR "Dying"[tiab] OR Fatal*[tiab] OR Mortalit*[tiab] OR "Mortality"[mh] OR "Postmortem"[tiab])))
Limit: Publication Type Exclude (Original)	NOT (“comment”[Publication Type] OR “editorial”[Publication Type] OR “review”[Publication Type] OR systematic[sb] OR "meta-analysis"[publication type] OR “systematic review”[tiab] OR “systematic literature review”[tiab] OR meta-analysis[tiab] OR "meta-analysis"[tiab] OR meta-analysis[tiab] OR "meta-analysis"[tiab] OR "pooled analysis"[tiab] OR “pooled analyses”[tiab] OR "pooled data"[tiab])
Limit: Exclude animal only	NOT ("Animals"[Mesh] NOT ("Animals"[Mesh] AND "Humans"[Mesh]))
Limit: Exclude child only	NOT (("infant"[Mesh] OR "child"[mesh] OR "adolescent"[mh]) NOT (("infant"[Mesh] OR "child"[mesh] OR "adolescent"[mh]) AND "adult"[Mesh]))
Limit: Date	From 1/3/2018

Search Strategy: CINAHL (S1: Original Research)

Database: CINAHL; Date of Search: 14/11/2024; 298 unique results
Terms searched in title or abstract

Set	Search Strategy
Physical activity (bouts)	((“intermittent activity” OR “intermittent exercise” OR "accumulated activity" OR bouts) AND ("Physical activity" OR "Exercise"))
Outcomes (Cardiovascular Disease Incidence OR Mortality OR Cardiometabolic Risk Factors OR Cardiorespiratory Fitness OR Frailty)	AND (((Arteriosclero* OR "Arteriosclerosis" OR Atherosclero* OR "Cerebral infarction" OR "Cerebrovascular diseases" OR "Cerebrovascular disease" OR "Coronary heart disease" OR "Heart failure" OR "Intracerebral Hemorrhage" OR "Intracerebral Hemorrhages" OR "Intracranial hemorrhage" OR "Intracranial hemorrhages" OR "Myocardial ischemia" OR "myocardial infarction" OR "Stroke" OR "Subarachnoid hemorrhage" OR "Subarachnoid hemorrhages" OR "Ischemic heart diseases" OR "Ischemic heart disease") AND ("risk" OR "risks" OR "Incidence" OR "incident" OR "Death" OR "Dying" OR Fatal* OR "Mortality" OR "Postmortem")) OR ("blood pressure" OR "systolic pressure" OR "diastolic pressure" OR "mean arterial" OR "bp response" OR "bp decrease" OR "bp reduction" OR "normotensive” OR “hypertension” OR “hypotension” OR “normotension” OR “hypertensive” OR “hypotensive” OR "Body weight" OR "Body composition" OR "Body Mass Index" OR “Waist circumference” OR "Body weight change" OR "Weight gain" OR "Weight status" OR "Overweight" OR "Weight Control" OR "Weight maintenance" OR "Weight regulation" OR "Weight stability" OR "Body composition" OR "Body Mass Index" OR “Waist circumference” OR "glucose intolerance" OR "glucose control" OR "insulin resistance" OR "prediabetes" OR "pre-diabetes" OR (diabetes AND ("type 2" OR "type II")) OR “lipoproteins” OR “cholesterol” OR “triglycerides” OR “triglyceride” OR “blood lipids” OR "lipoprotein") OR ("Cardiorespiratory fitness" OR "VO2 max" OR "maximal oxygen uptake" OR "peak oxygen uptake" OR "aerobic capacity") OR ((“mortality” OR “frailty”) AND ("risk" OR "risks" OR "Incidence" OR "incident" OR "Death" OR "Dying" OR Fatal* OR "Mortality" OR "Postmortem")))
Original Research	NOT (“systematic review” OR “systematic literature review” OR metaanalysis OR "meta analysis" OR metanalyses OR "meta analyses"" OR "pooled analysis" OR “pooled analyses” OR "pooled data")
Limits	English language<br>Peer reviewed<br>Exclude Medline records<br>Human<br>From 1/3/2018

Search Strategy: Cochrane (S1: Original Research)

Database: Cochrane; Date of Search: 14/11/2024; 602 results
Terms searched in title, abstract, or keywords

Set	Search Strategy
Physical activity (bouts)	((“intermittent activity” OR “intermittent exercise” OR "accumulated activity" OR bouts) AND ("Physical activity" OR "Exercise"))
Outcomes (Cardiovascular Disease Incidence OR Mortality OR Cardiometabolic Risk Factors OR Cardiorespiratory Fitness OR Frailty)	AND (((Arteriosclero* OR "Arteriosclerosis" OR Atherosclero* OR "Cerebral infarction" OR "Cerebrovascular diseases" OR "Cerebrovascular disease" OR "Coronary heart disease" OR "Heart failure" OR "Intracerebral Hemorrhage" OR "Intracerebral Hemorrhages" OR "Intracranial hemorrhage" OR "Intracranial hemorrhages" OR "Myocardial ischemia" OR "myocardial infarction" OR "Stroke" OR "Subarachnoid hemorrhage" OR "Subarachnoid hemorrhages" OR "Ischemic heart diseases" OR "Ischemic heart disease") AND ("risk" OR "risks" OR "Incidence" OR "incident" OR "Death" OR "Dying" OR Fatal* OR "Mortality" OR "Postmortem")) OR ("blood pressure" OR "systolic pressure" OR "diastolic pressure" OR "mean arterial" OR "bp response" OR "bp decrease" OR "bp reduction" OR "normotensive" OR “hypertension” OR “hypotension” OR “normotension” OR “hypertensive” OR “hypotensive” OR "Body weight" OR "Body composition" OR "Body Mass Index" OR “Waist circumference” OR "Body weight change" OR "Weight gain" OR "Weight status" OR "Overweight" OR "Weight Control" OR "Weight maintenance" OR "Weight regulation" OR "Weight stability" OR "Body composition" OR "Body Mass Index" OR “Waist circumference” OR "glucose intolerance" OR "glucose control" OR "insulin resistance" OR "prediabetes" OR "pre-diabetes" OR (diabetes AND ("type 2" OR "type II")) OR “lipoproteins” OR “cholesterol” OR “triglycerides” OR “triglyceride” OR “blood lipids” OR "lipoprotein") OR ("Cardiorespiratory fitness" OR "VO2 max" OR "maximal oxygen uptake" OR "peak oxygen uptake" OR "aerobic capacity") OR ((“mortality” OR “frailty”) AND ("risk" OR "risks" OR "Incidence" OR "incident" OR "Death" OR "Dying" OR Fatal* OR "Mortality" OR "Postmortem")))
Limits	Trials<br>Word variations will not be searched<br>From March 2018

Search Strategy: PubMed (S2: Systematic Reviews, Meta-Analyses, Pooled Analyses, and High-Quality Reports)

Database: PubMed; Date of Search: 14/11/2024; 225 results

Set	Search Strategy
Physical activity	(("Activity bouts"[tiab] OR "Daily steps"[tiab] OR "High intensity activity"[tiab] OR "Interval training"[tiab] OR "Pedometer"[tiab] OR "Step count"[tiab] OR "Steps/day"[tiab] OR "high intensity interval training"[tiab]) OR ((("High intensity"[tiab] AND "training"[tiab]) OR "Interval training"[tiab] OR "Pedometer"[tiab]) NOT medline[sb]))
Limit: Publication Type Include (Systematic Reviews/MetaAnalyses)	AND (systematic[sb] OR meta-analysis[pt] OR review[tiab] OR "systematic review"[tiab] OR "systematic literature review"[tiab] OR meta-analysis[tiab] OR "meta analysis"[tiab] OR metanalyses[tiab] OR "meta analyses"[tiab] OR "pooled analysis"[tiab] OR "pooled analyses"[tiab] OR "pooled data"[tiab])
Limit: Publication Type Exclude (Systematic Reviews/MetaAnalyses)	NOT ("comment"[Publication Type] OR "editorial"[Publication Type])
Limit: language	AND (English[lang])
Limit: Exclude animal only	NOT ("Animals"[Mesh] NOT ("Animals"[Mesh] AND "Humans"[Mesh])) NOT (("infant"[Mesh] OR "child"[mesh] OR "adolescent"[mh])
Limit: Exclude child only	NOT (("infant"[Mesh] OR "child"[mesh] OR "adolescent"[mh]) AND "adult"[Mesh]))
Limit : Date	From 5/4/2017

Search Strategy: CINAHL (S2: Systematic Reviews, Meta-Analyses, Pooled Analyses, and High-Quality Reports)

Database: CINAHL; Date of Search: 14/11/2024; 281 unique results
Terms searched in title or abstract

Set	Search Strategy
Physical activity	("Activity bouts" OR "Daily steps" OR "High intensity activity" OR "Interval training" OR Pedometer OR "Step count" OR "Steps/day" OR 'high intensity interval training" OR ("High intensity" AND "training"))
Systematic Reviews and Meta-Analyses	AND (“systematic review” OR “systematic literature review” OR review OR metaanalysis OR "meta analysis" OR metanalyses OR "meta analyses"" OR "pooled analysis" OR “pooled analyses” OR "pooled data")
Limits	English language<br>Peer reviewed<br>Exclude Medline records<br>Human<br>From 5/4/2017

Search Strategy: Cochrane (S2: Systematic Reviews, Meta-Analyses, Pooled Analyses, and High-Quality Reports)

Database: Cochrane; Date of Search: 14/11/2024; 14 results
Terms searched in title, abstract, or keywords

Set	Search Strategy
Physical activity	("Activity bouts" OR "Daily steps" OR "High intensity activity" OR "Interval training" OR Pedometer OR "Step count" OR "Steps/day" OR "high intensity interval training" OR ("High intensity" AND training))
Limits	Word variations not searched<br>Cochrane Reviews and Other Reviews<br>From April 2017
