# Supplementary material for: Synthesis and Validity of Accelerometer Devices and Methods Used in Epidemiological Studies of Physical Activity Bout Duration and Health Outcomes: A Systematic Review
Source: Sports Med Open. 2026 Jul 1;12:84. doi: 10.1186/s40798-026-01039-4 (PMC13323697; doi:10.1186/s40798-026-01039-4)
Supplement: Supplementary file 3 — Supplementary Material 3. [file 40798_2026_1039_MOESM3_ESM.pdf]

Supplementary file 3: Inclusion/Exclusion Criteria

Category	*Inclusion/Exclusion Criteria*
Publication Language	*Include*:<br>- Studies published with full text in English
Publication Status	*Include*:<br>- Studies published in peer-reviewed journals<br>- Reports determined to have appropriate suitability and quality<br>*Exclude*:<br>- Grey literature, including unpublished data, manuscripts, abstracts, conference proceedings
Research Type	*Include*:<br>- Original research<br>- Meta-analyses<br>- Systematic reviews
Study Subjects	*Include*:<br>- Human subjects
Age of Study Subjects	*Include*:<br>- 18 years of age and above
Health Status of Study Subjects	*Include*:<br>- Only studies conducted in general population<br>*Exclude*:<br>- Studies on patients with existing cardiovascular diseases (CVD)<br>- Studies on high performance athletes
Comparison	*Include* studies in which the comparison is:<br>- Adults exposed to different doses of physical activity
Date of Publication	*Include*:<br>- 1/3/2018 to present (S1)<br>- 5/4/2017 to present (S2)
Study Design / Type of research	*Include*:<br>- Original research articles<br>- Intervention studies<br>- Longitudinal studies<br>- Cross-sectional studies
Size of Study Groups	*Include*:<br>- All
Intervention/ Exposure	*Include*:<br>- Intervention or observational studies that use accelerometers or other objective measures to assess physical activity performed in short bouts (bouts should be spread throughout the day, but not within the same session of exercise).<br>- Studies with any bout (duration ideally less than 10 minutes)<br>- For intervention studies, the duration of the physical activity exposure should be at least 12 weeks.<br>*Exclude*:<br>- Studies examining the metabolic response (e.g., insulin sensitivity, lipid values) to a single dose of physical activity or acute bouts.<br>- Exposure measured by a single measure of physical fitness (cardiovascular fitness, strength, flexibility, walking speed in older adults): where the measure of physical activity is based only on physical fitness measures (single or combined variables)<br>- Studies that do not include physical activity (or the lack thereof) as the primary exposure variable or use solely as a confounding variable **and**

	**training/exercise studies that do not assess free-living PA as an exposure.**<br>- Studies of a specific therapeutic exercise (range of motion exercise, inspiratory muscle training)
Outcome	*Include studies in which the outcome is*:<br>- All-causes and CVD mortality<br>- CVD<br>- Type 2 diabetes<br>- Cardio metabolic risk factors (blood pressure, blood lipids [total cholesterol, HDL-cholesterol, LDL-cholesterol, triglycerides], body mass, BMI, waist circumference)<br>- Cardiorespiratory fitness<br>- **Frailty**<br>*Exclude*:<br>- Congenital heart disease<br>- Studies on progression of CVD
Multiple Publications of Same Data	*Exclude*:<br>No restriction

Note: criteria in bold have been added to the criteria used by PAGAC.
