# Supplementary material for: Synthesis and Validity of Accelerometer Devices and Methods Used in Epidemiological Studies of Physical Activity Bout Duration and Health Outcomes: A Systematic Review
Source: Sports Med Open. 2026 Jul 1;12:84. doi: 10.1186/s40798-026-01039-4 (PMC13323697; doi:10.1186/s40798-026-01039-4)
Supplement: Supplementary file 4 — Supplementary Material 4. [file 40798_2026_1039_MOESM4_ESM.pdf]

Supplementary file 4: Risk of bias. Modified QUADAS-2 tool.

Criteria items
Domain 1: Patient selection/Study design
1. Did the study include a range of physical activity bouts? (i.e., different types - at least 2 - of physical activity bouts and/or of different intensity and duration particularly if only one type of physical activity is studied)
2. Did the study protocol include free-living measurement or at least one part/activity with natural transitions (i.e., activities performed without fixed order of instructions) and following an intermittent pattern?
3. Did the study provide any information about the inclusion/exclusion of the recruiting process?
4. Did the study include an adequate number of participants? (i.e., sample size calculated based on previously published or pilot study data, or a sample of convenience ≥ 45 participants?)
Domain 2: Index measure
5. Was the algorithm of the validated outcome reported (i.e., formula, features description, epoch used), or was at least further information cited?
Domain 3: Criterion measure
6. Is the selected reference the gold standard?
Domain 4: Flow and timing
7. Did the authors provide any information about data synchronization between index and reference measures?
8. Were all participants included in the analyses or were any exclusion reasons provided?

Legend. The risk of bias for each article was evaluated using the Quality Assessment of Diagnostic Accuracy Studies (QUADAS-2) tool. The tool comprises four domains (i.e., patient selection, index measure, criterion measure, and flow/timing). Following the QUADAS-2 guidelines, we selected a set of signaling questions for each domain and added questions modified from the QUADAS-2 background document based on core principles, recommendations, and expert statements for validation studies
