# Supplementary figures and images for: Synthesis and Validity of Accelerometer Devices and Methods Used in Epidemiological Studies of Physical Activity Bout Duration and Health Outcomes: A Systematic Review
Source: Sports Med Open. 2026 Jul 1;12:84. doi: 10.1186/s40798-026-01039-4 (PMC13323697; doi:10.1186/s40798-026-01039-4)

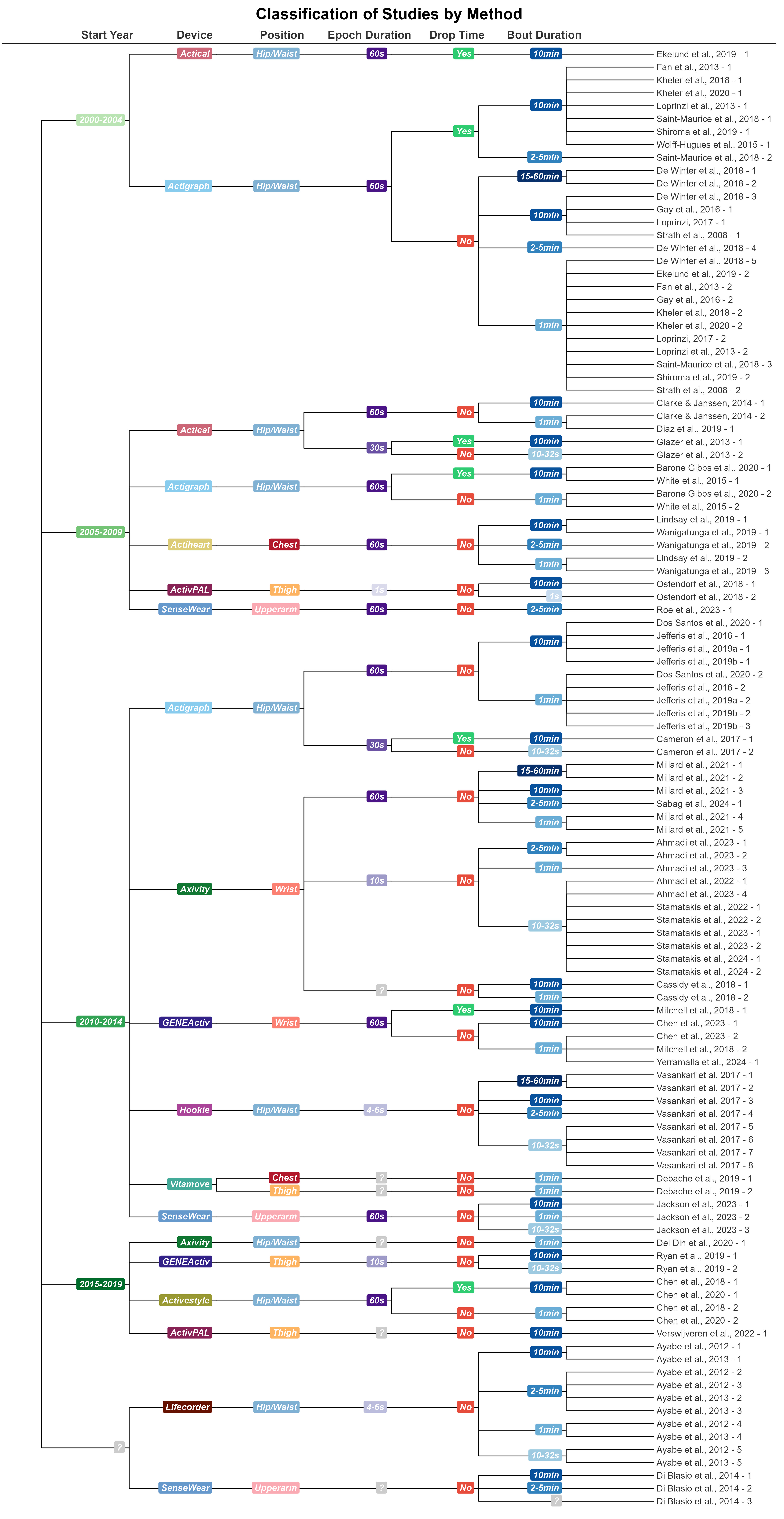

Supplement: Supplementary file 5 — Supplementary Material 5. [file 40798_2026_1039_MOESM5_ESM.jpeg]
