# Supplementary material for: Synthesis and Validity of Accelerometer Devices and Methods Used in Epidemiological Studies of Physical Activity Bout Duration and Health Outcomes: A Systematic Review
Source: Sports Med Open. 2026 Jul 1;12:84. doi: 10.1186/s40798-026-01039-4 (PMC13323697; doi:10.1186/s40798-026-01039-4)
Supplement: Supplementary file 6 — Supplementary Material 6. [file 40798_2026_1039_MOESM6_ESM.pdf]

Supplementary file 6: Pairwise Dependencies Between Methodological Parameters.

Variable 1	Variable 2	Adjusted p-Value	Cramér's V
Position	Epoch Duration	<.001	.50
Position	Drop Time	.156	.27
Position	Bout Duration	.106	.33
Position	Device	<.001	.93
Position	Start Year	<.001	.46
Epoch Duration	Drop Time	.021	.35
Epoch Duration	Bout Duration	<.001	.48
Epoch Duration	Device	<.001	.73
Epoch Duration	Start Year	<.001	.46
Drop Time	Bout Duration	<.001	.53
Drop Time	Device	.023	.43
Drop Time	Start Year	.003	.37
Bout Duration	Device	<.001	.41
Bout Duration	Start Year	.015	.31
Device	Start Year	<.001	.79

Legend. This table presents the adjusted p-values (Benjamini–Hochberg procedure) and effect sizes (Cramér's V) resulting from Fisher's exact tests with Monte Carlo simulation, conducted to assess the associations between each pair of categorical methodological parameters. A significance threshold of $p < .05$ was applied. Cramér's V is interpreted using Cohen's thresholds: negligible (< 0.1), small (0.1–0.29), medium (0.3–0.49), large (0.5–0.69), very large (0.7–0.89), and extremely large (≥ 0.9).
