# Supplementary material for: Synthesis and Validity of Accelerometer Devices and Methods Used in Epidemiological Studies of Physical Activity Bout Duration and Health Outcomes: A Systematic Review
Source: Sports Med Open. 2026 Jul 1;12:84. doi: 10.1186/s40798-026-01039-4 (PMC13323697; doi:10.1186/s40798-026-01039-4)
Supplement: Supplementary file 7 — Supplementary Material 7. [file 40798_2026_1039_MOESM7_ESM.pdf]

Supplementary file 7: Characteristics of validation studies identified in the systematic review.

Validation study	*Native epidemiological study*	*Study aim*	*D1: target population*	*D2: criterion measure (type , location, epoch)*	*D3: index measure (type, location, epoch length)*	*D4: testing conditions*	*D5: processing*	*D5 (continued): bout definition FITT*	*D6: statistical analysis*	*Main results for bout detection*
Brage et al., 2003	[b]	To define methods for estimating VO2, speed and step frequency.	22 healthy participants (22m)	Oxygen uptake estimation using indirect calorimetry (EO Sprint, NA, 30s)	CSA (uniaxial accelerometer, hip/waist-worn, NK)	*Setting*: Laboratory<br>*Protocol*: Two sessions of two treadmill walking bouts and eight treadmill running bouts (5min) at different speeds (3-20km.h) in the laboratory.	*Criterion data*: Averaged VO2 values over the final 90s of each stage were considered to represent each speed.<br>*Index data*: Averaged counts per minute over the final 4min of each stage were considered to represent each speed.<br>*Method*: Prediction models were defined using multiple linear regression model.<br>*Sync*: NK	F: not defined<br>I: VO_2 prediction models<br>T: not defined<br>T: walking and running activities	ANOVA<br>R^2	NA
Brage et al., 2005	Lindsay et al., 2019	To define a method for estimating activity energy expenditure	20 participants (9w/11m)	Energy expenditure estimation using indirect calorimetry (Cosmed K4b2, NA, breath-by-breath)	Actiheart (multisensor device with uniaxial accelerometer, chest, 15s)	*Setting*: Laboratory<br>*Protocol*: Three treadmill walking bouts and three treadmill running bouts (4min) at different speeds (3.2-12.1km.h) in the laboratory.	*Criterion data*: After smoothing and filtering, energy expenditure was calculated using VO2 and VCO2 values. Measured resting metabolic rate was removed from averaged values over the final last minute of each stage to represent the net energy expenditure of each speed.<br>*Index data*: Averaged counts and heart rate over the final last 45s of each stage were considered to represent each speed.<br>*Method*: Prediction model was defined using multiple linear regression model.<br>**Sync**: Yes	F: not defined<br>I: energy expenditure prediction model using acceleromter counts and heart rate<br>T: not defined<br>T: walking and running activities	R^2<br>SEE<br>ICC<br>Paired T-test	NA

Colley et Tremblay, 2011	Clarke and Janssen, 2014 / Ekelund et al., 2019	To define cut-points for estimating physical activity intensity	38 underage and adult participants	Physical activity intensity estimation using the Compendium of Physical Activities (NA, NA, NA)	Actical (omnidirectionnal accelerometer, NK, 60s)	*Setting*: Laboratory<br>*Protocol*: Two treadmill walking bouts and one treadmill running bout (6min) at different speeds (3-8km.h) in the laboratory.	*Criterion data*: METs values for each speed were identified using the Compendium of Physical Activities.<br>*Index data*: First and last minutes of each bout were ignored then the averaged counts per minute over the bout were considered to represent each speed.<br>*Method*: Cut-points were defined using a decision boundary analytical approach.<br>*Sync*: NA.	F: not defined<br>I: MPA >1535cpm, VPA >3960cpm (for adults)<br>T: not defined<br>T: walking activity	Sensitivity and specificity of cut-points (tested on the same dataset)	NA
Copeland et Esliger, 2009	Jefferis et al., 2016 / Dos Santos et al., 2020 / Jefferis et al., 2019a / Jefferis et al., 2019b	To define cut-points for estimating physical activity intensity	38 older healthy participants (20w/18m, 69.7yrs)	Oxygen uptake estimation using Vista Mini CPX (indirect calorimetry, NA, 30s)<br>Physical activity intensity estimation using the Compendium of Physical Activities (NA, NA, NA)	Actigraph 7164 (uniaxial accelerometer, hip/waist-worn, 60s)	*Setting*: Laboratory<br>*Protocol*: Three treadmill walking bouts (6min) at different speeds (2.4-4.8km.h) in the laboratory.	*Criterion data*: First minutes of each bout were ignored then the averaged VO2 values were considered to represent each speed.<br>*Index data*: Averaged counts per minute over each bout were considered to represent each speed.<br>*Method*: Cut-point was defined using averaged counts corresponding to the walk at 3.2 km.h.<br>*Sync*: NK.	F: not defined<br>I: MPA >1041cpm<br>T: not defined<br>T: walking activity	NA	NA
Crouter et al., 2013	Saint-Maurice et al., 2018	To test methods for estimating physical activity intensity	29 participants (17w/12m, 38yrs)	Oxygen uptake estimation using Cosmed K4b2 (indirect calorimetry, NA, breath-by-breath)	ActiGraph GT1M (uniaxial accelerometer, hip/waist-worn, 1s)	*Setting*: Free-living<br>*Protocol*: 5-6h period outside the laboratory (work and leisure activities).	*Criterion data*: VO2 values were averaged over 1min epoch and then expressed in METs.<br>*Index data*: Counts per minute were averaged over 10s and 1min epochs depending on the method used. Four methods were then applied (2010 Crouter algorithm / 2006 Crouter algorithm / NHANES cut-points / Matthews cut-points).<br>*Method*: Methods were tested	F: not defined<br>I: 2010 Crouter algorithm / 2006 Crouter algorithm / NHANES cut-points / Matthews cut-points<br>T: not defined<br>T: daily physical activities	Pairwise comparisons with Bonferroni adjustments Mean error score and 95% prediction interval	NA

							using indirect calorimetry comparison.<br>*Sync*: Yes (digital clock).			
Crouter et Basset, 2008	Glazer et al., 2013	To define a model for estimating physical activity intensity	48 participants (24w/24m, 35yrs)	Oxygen uptake estimation using Cosmed K4b2 (indirect calorimetry, NA, breath-by-breath)	Actical (omnidirectionnal accelerometer, hip/waist-worn, 15s)	*Setting*: Semi free-living<br>*Protocol*: Eighteen lifestyle and sporting activities (10min - sedentary activities, household activities, locomotive activities).	*Criterion data*: First and last minutes of each activity were ignored then the averaged VO2 values were considered to represent each activity. Next, values were expressed in METs.<br>*Index data*: First and last minutes of each activity were ignored then counts per minute and coefficients of variation were averaged over 1min epoch.<br>*Method*: Prediction model was defined using a 2-regression model (according to the CV of four consecutive 15s epochs) with an inactivity threshold.<br>*Sync*: Yes (digital clock).	F: not defined<br>I: METs prediction model<br>T: ≥1min<br>T: daily physical activities	Cross-validation (Mean, rMSE)<br>R^2<br>APE / Kappa statistics	NA

Esliger et al., 2011	Mitchell et al., 2018	To define cut-points for estimating physical activity intensity	60 healthy participants (37w/23m, 49.4yrs)	Oxygen uptake estimation using Cosmed K4b2 (indirect calorimetry, NA, breath-by-breath)	GENEA (triaxial accelerometer, wrist and hip/waist-worn, 1s)	*Setting*: Semi free-living<br>*Protocol*: Twelve semi-structured activities (4.5min) in and outside the laboratory (sedentary activities, household activities, locomotive activities).	*Criterion data*: VO2 values were averaged over 1min epoch then steady-state values were visually selected to represent each activity. Next, values were expressed in METs.<br>*Index data*: Raw data were summarized into signal magnitude vector over 1min epoch then values corresponding to criterion steady-state were selected to represent each activity.<br>*Method*: Cut-points were defined using ROC curve analysis.<br>*Sync*: Yes (GMT server and custom spreadsheet applications).	F: not defined<br>I: left wrist LIPA 217–644, MPA 645–1810, VPA >1810 / right wrist LIPA 386–439, MPA 440–2098, VPA >2098 / waist LIPA 77–219, MPA 645–1810, VPA >2056<br>T: not defined<br>T: daily physical activities	Pearson correlations Receiver operating characteristic curve analyses	NA
Freedson et al., 1998	[b] / White et al., 2015 / Dos Santos et al., 2020 / Ekelund et al., 2019 / Shiroma et al., 2019 / Freedson et al., 1998	To define cut-points for estimating physical activity intensity	50 participants (25w/25m)	Oxygen uptake estimation using indirect calorimetry (open circuit spirometry with a computer-based data acquisition system, NA, 60s)	CSA (uniaxial accelerometer, hip/waist-worn, 60s)	*Setting*: Laboratory<br>*Protocol*: Two treadmill walking bouts and one treadmill running bout (6min) at different speeds (4.8-9.7km.h) in the laboratory.	*Criterion data*: Averaged VO2 values over the final 3min of each stage were considered to represent each speed. Then, values were expressed in METs.<br>*Index data*: Averaged counts per minute over each stage were considered to represent each speed.<br>*Method*: cut-points were defined using indirect calorimetry comparison (linear regression).<br>*Sync*: Yes (external timepiece)	F: not defined<br>I: LIPA <1952cpm / MPA 1952-5724cpm / VPA 5725-9498 / Very hard >9498cpm<br>T: not defined<br>T: walking and running activities	One-way repeated measures ANOVA Cross-validation (mean differences, SEE)	NA

Heil, 2006	Clarke and Janssen, 2014 / Glazer et al., 2013	To define a model for estimating activity energy expenditure and cut-points for estimating physical activity intensity	48 underage and adult participants (22w/26m)	Energy expenditure estimation using indirect calorimetry (VmaxST™, NA, NK)	Actical (omnidirectionnal accelerometer, wirst, ankle and hip/waist-worn, 60s)	*Setting*: Semi free-living<br>*Protocol*: Ten semi-structured activities (3 to 5min) in the laboratory (sedentary activities, household activities, locomotive activities).	*Criterion data*: First minutes of each activity were ignored then the averaged VO2 and VCO2 values after removing measured resting metabolic rate were considered to represent the net energy expenditure of each activity.<br>*Index data*: First minutes of each activity were ignored then the averaged counts per minute were considered to represent each activity.<br>*Method*: Activity energy expenditure models and cut-points were defined using either a single regression equation or two nonoverlapping linear regression equations and were location specific.<br>*Sync*: Yes (digital clock).	F: not defined<br>I: location specific cut-points and energy expenditure prediction equations<br>T: ≥1min / ≥3min / ≥5 min<br>T: daily physical activities	R^2 and SEE<br>Intraclass reliability and test-retest reliability<br>Correlation coefficient	NA
Hildebrand et al., 2014	Ahmadi et al., 2022 / Ahmadi et al., 2023 / Cassidy et al., 2018 / Stamatakis et al., 2022 / Stamatakis et al., 2023 / Millard et al., 2021 / Sabag et al., 2024 / Chen et al., 2023 / Yerramalla et al., 2024 / Stamatakis	To define cut-points for estimating physical activity intensity	60 underage and adult healthy participants (29w/31m)	Oxygen uptake estimation using indirect calorimetry (VMax Encore, NA, NK)	ActiGraph GT3X+ (triaxial accelerometer, hip/waist and wrist-worn , 1s) GENEActiv (triaxial, hip and wrist-worn, 1s).	*Setting*: Semi free-living<br>*Protocol*: Eight semi-structured activities (4.5min) in the laboratory (sedentary activities, household activities, locomotive activities).	*Criterion data*: First and last minutes of each activity were ignored, then VO2 values were expressed in METs.<br>*Index data*: First and last minutes of each activity were ignored then the averaged vector magnitude and ENMO values after data reduction (averaging values per 1s epoch) were considered to represent each activity.<br>*Method*: Cut-points were defined using linear regression models.<br>*Sync*: Yes.	F: not defined<br>I: ActiGraph hip MVPA >69.1mg, VPA >258.7mg / ActiGraph wrist MVPA>100.6mg, VPA >428.8mg / GENEActiv hip MVPA >68.7mg, VPA >266.8mg / GENEActiv wrist MVPA >93.2mg, VPA >418.3mg (for adults)<br>T: not defined<br>T: daily physical activities	R^2<br>10–cross-validation mode (leave-one-out cross-validation)	NA

	et al., 2024									
Hooker et al., 2011	Diaz et al., 2019	To define a model for estimating activity energy expenditure and cut-points for estimating physical activity intensity	73 participants (43w/30m)	Energy expenditure estimation using indirect calorimetry (Cosmed K4b2, NA, breath-by-breath)	Actical (omnidirectionnal accelerometer, hip/waist-worn, 60s)	*Setting*: Semi free-living<br>*Protocol*: Eight semi-structured activities (4 to 5min) in the laboratory (sedentary activities, household activities, locomotive activities).	*Criterion data*: First minutes of each activity were ignored. For physical activity intensity, VO2 values were expressed in METs. For activity energy expenditure, energy expenditure was calculated using VO2 and VCO2 values, then measured resting metabolic rate was removed to represent the net energy expenditure of each activity.<br>*Index data*: Averaged counts per minute over the final 2min of each bout were considered to represent each activity.<br>*Method*: Activity energy expenditure models and cut-points were defined using non-linear regression models.<br>*Sync*: NK.	F: not defined<br>I: MPA >1065cpm<br>T: not defined<br>T: daily physical activities	Receiver operating characteristic curve analyses<br>Sensitivity, specificity, and positive predictive value<br>R^2	NA

Jakicic et al., 2004	Jackson et al., 2023	To test a physical activity monitor for estimating energy expenditure	40 participants (20w/20m, 23.2yrs)	Energy expenditure estimation using indirect calorimetry (Parvomedics or SensorMedics Vmax, NA, 60s)	SenseWear Pro Armband (bi-axial accelerometer, upper-arm-worn, 60s)	*Setting*: Laboratory<br>*Protocol*: Four standardised activities (20 to 30min) in the laboratory (including cycling).	*Criterion data*: Energy expenditure was computed by multiplying the VO2 values by the caloric equivalent based on the RER, then summed over exercise bout to represent the energy expenditure of each activity.<br>*Index data*: Energy expenditure during each exercise was estimated either using a generalized proprietary algorithm developed by the manufacturer, or using exercise-specific algorithms developed specifically for this study by the manufacturer.<br>*Method*: Monitor was tested using doubly labeled water comparison.<br>*Sync*: NK	F: not defined<br>I: monitor calculated or with energy expenditure prediction algorithm<br>T: not defined<br>T: exercise (treadmill walking, stair stepping, cycle ergometry and arm ergometry)	ICC<br>Two-factor repeated measures ANOVA<br>Bland–Altman plots	NA
Kumahara et al., 2004	Ayabe et al., 2012, Ayabe et al., 2013	To define a method for estimating physical activity intensity	10 healthy participants (10m, 24.4yrs)	Oxygen uptake estimation using indirect calorimetry (Douglas bag, NA, 60s)	Lifecorder (uniaxial accelerometer, hip/waist-wornn, 4s)	*Setting*: Laboratory<br>*Protocol*: Six treadmill walking bouts and three treadmill running bouts (4min) at different speeds (2.4-9.6km.h) in the laboratory.	*Criterion data*: Averaged VO2 values over the final minute of each bout were considered to represent each activity.<br>*Index data*: Activity levels were directly calculated by the monitor (0·0, 0·5, and 1·0–9·0;) and converted by an algorithm to calculate EE (kcal).<br>*Method*: Prediction model was defined using a quadratic curvilinear regression equation.<br>*Sync*: NK.	F: not defined<br>I: categorized into 1-9 activity levels by the monitor, then MPA 4-6 / VPA 7-9<br>T: not defined<br>T: walking and running activities	One-way ANOVA	NA

Leenders et al., 2001	[b]	To test physical activity monitors for estimating energy expenditure	13 healthy participants (13w, 25.8yrs)	Energy expenditure estimation using doubly labeled water (NA, NA, NA)	Tritrac (triaxial accelerometer, hip/waist-worn, 60s)<br>CSA (uniaxial accelerometer, hip/waist-worn, 60s)<br>Yamax-Digiwalker-500 (pedometer, hip/waist-worn, NA)	*Setting*: Free-living<br>*Protocol*: Seven days of usual activities outside the laboratory.	*Criterion data*: Activity energy expenditure values were calculated by removing energy expenditure due to the thermic effect of meals (estimation) and measured resting metabolic rate.<br>*Index data*: Tritrac - energy expenditure was calculated from vector magnitude data by using the manufacturer's proprietary equations. CSA - energy expenditure was calculated from activity counts using a formula. Yamax - energy expenditure was calculated from daily steps by the manufacturer's proprietary regression equation.<br>*Method*: Monitors were tested using doubly labeled water comparison and linear regression analysis was conducted.<br>*Sync*: NA/NK?	F: not defined<br>I: not defined - all intensities were considered without distinction<br>T: not defined - all durations were considered without distinction<br>T: daily physical activities	Pearson's correlation coefficient<br>Paired t-tests	NA
Mackey et al., 2011	Roe et al., 2019	To test a physical activity monitor for estimating energy expenditure	19 older participants (8w/11m, 82.0yrs)	Energy expenditure estimation using doubly labeled water (NA, NA, NA)	SenseWear Pro Armband (multisensor device with bi-axial accelerometer, upper-arm-worn, 60s)	*Setting*: Free-living<br>*Protocol*: Approximately two weeks of usual activities outside the laboratory.	*Criterion data*: Activity energy expenditure values were calculated by removing energy expenditure due to the thermic effect of meals (estimation) and measured resting metabolic rate from total energy expenditure.<br>*Index data*: Total energy exenditure was directly estimated by the monitor. Then, activity energy expenditure values were calculated by removing energy expenditure due to the thermic effect of meals (estimation) and measured resting metabolic rate.	F: not defined<br>I: not defined - all intensities were considered without distinction<br>T: not defined - all durations were considered without distinction<br>T: daily physical activities	Paired t tests<br>Pearson correlation coefficients<br>ICC two-way ANOVA<br>Bland–Altman plots	NA

							Method: Monitor was tested using doubly labeled water comparison and linear regression analysis was conducted.<br>*Sync*: NA			
Matthews, 2005	Saint-Maurice et al., 2018 / Strath et al., 2008	To review monitor calibration studies and to define new cut-points for estimating physical activity intensity[a]	NK	Multiple (authors proposed a cutpoint for MVPA which was cross-validated from the data of two other studies)	Actigraph CSA 7164 (uniaxial, hip/waist-worn, 60s)	Multiple (authors proposed a cutpoint for MVPA which was cross-validated from the data of two other studies)	Multiple (authors proposed a cutpoint for MVPA which was cross-validated from the data of two other studies)	F: not defined<br>I: MPA >760cpm<br>T: not defined<br>T: daily physical activities	Cross-validation	NA

Ohkawara et al., 2011	Chen et al., 2018 / Chen et al., 2020	To define methods for estimating physical activity intensity	66 participants (35w/31m, 42.4yrs)	Energy expenditure estimation using indirect calorimetry (Douglas bag, NA, NK)	LIS3LV02DQ (triaxial accelerometer, hip/waist-worn, 10s)	*Setting*: Semi free-living<br>*Protocol*: Fourteen semi-structured activities (3 to 7min) in the laboratory (sedentary activities, household activities, locomotive activities).	*Criterion data*: First minutes of each activity were ignored. Energy expenditure was calculated using VO2 and VCO2 values, then METs were calculated as the energy expenditure of each activity divided by the measured resting metabolic rate.<br>*Index data*: After filtering, vector magnitude values were processed over 10s epoch to determine various output variables and were considered to represent each activity. Ratio between filtered synthetic acceleration and unfiltered synthetic acceleration were used to classify household and locomotive activities.<br>*Method*: Prediction models were defined using linear and non-linear regression models.<br>*Sync*: NK.	F: not defined<br>I: METs prediction algorithm<br>T: not defined<br>T: daily physical activities	Pearson's correlation coefficient<br>SEE<br>One-way ANOVA	NA
St-Onge et al., 2007	Jackson et al., 2023	To test a physical activity monitor for estimating energy expenditure	45 participants (32w/13m, 35.1yrs)	Energy expenditure estimation using doubly labeled water (NA, NA, NA)	HealthWear Bodymedia (multisensor device with bi-axial accelerometer, upper-arm-worn, NK)	*Setting*: Free-living<br>*Protocol*: Approximately ten days of usual activities outside the laboratory.	*Criterion data*: Activity energy expenditure values were calculated by removing energy expenditure due to the thermic effect of meals (estimation) and measured resting metabolic rate total energy expenditure..<br>*Index data*: Activity energy expenditure values were directly estimated by the device.<br>*Method*: Monitor was tested using doubly labeled water comparison and linear regression analysis was conducted.<br>*Sync*: NA	F: not defined<br>I: not defined - all intensities were considered without distinction<br>T: not defined - all durations were considered without distinction<br>T: daily physical activities	Paired t tests<br>ICC one-factor random effect<br>Bland–Altman plots	NA

Troaino et al., 2008	Cameron et al., 2017 / Gay et al., 2016 / Fan et al., 2013 / Kheler et al., 2018 / Loprinzi et al., 2013 / Loprinzi, 2017 / Wolff-Hugues et al., 2015 / De Winter et al., 2018 / Dos Santos et al., 2020 / Kehler et al. 2020	To define cut-points for estimating physical activity intensity[a]	7176 participants	NA	Actigraph 7164 (uniaxial accelerometer, hip/waist-worn, 60s)	*Setting*: Free-living *Protocol*: Seven days of usual activities outside the laboratory.	*Criterion data*: NA *Index data*: Time spent in activity of a defined intensity was determined by summing minutes in a day where the count met the criterion for that intensity. *Method*: Cut-points were calculated as a weighted average of criteria determined from four studies (Brage et al., 2003, Freedson et al., 1998, Leenders et al., 2001, Yngve et al., 2003). *Sync*: NK.	F: not defined I: MPA >2020cpm / VPA >5999cpm T: ≥1min, ≥10min* T: daily physical activities * "with allowance for interruptions of 1 or 2 min below threshold"	NA	NA

Vaha-Ypya et al., 2015	Vasankari et al. 2017	To define cut-points for estimating physical activity intensity	29 healthy participants (14w/15m, 35yrs)	Oxygen uptake estimation using indirect calorimetry (Oxycon, NA, breath-by-breath)	Hookie AM20 (triaxial accelerometer, hip/waist-worn, 6s)	*Setting*: Laboratory<br>*Protocol*: Incremental walking/running exercise on a 200m oval indoor track (from 0.6 m.s, increased by 0.4 m.s at every 2.5 minutes until volitional exhaustion).	*Criterion data*: Averaged VO2 values over the final 2min of each stage were considered to represent each speed. Then, values were expressed in METs.<br>*Index data*: Mean amplitude deviation (MAD) values over 6s epoch for the final 2min of each stage were considered.<br>*Method*: VO2 prediction model was defined using both gamma regression and linear regression models. Cut-points were defined using ROC curve analysis.<br>*Sync*: NK.	F: not defined<br>I: MPA >91mg / VPA >414mg (from MAD values)<br>T: not defined<br>T: walking and running activities	Pearson's correlation coefficient Receiver operator characteristics curves analysis	NA
Welk et al, 2007	Di Blasio et al., 2014 / Saint-Maurice et al., 2018	To test methods and monitors for estimating physical activity intensity[c]	30 participants (17w/13m, 24.9yrs)	Energy expenditure estimation using a portable gait analysis system (IDEEA monitor, both feet - both thighs - chess, 1s)	SenseWear Pro2 (bi-axial accelerometer, upper-arm-worn, 60s)<br>Actigraph MTI (uniaxial accelerometer, hip/waist-worn, 60s)	*Setting*: Free-living<br>*Protocol*: One day of usual activities outside the laboratory.	*Criterion data*: Energy expenditure values are directly exported in 1min epochs. Then, values were expressed in METs.<br>*Index data*: Raw data from both monitors were exported in 1min epoch. For SP2 data, an energy expenditure estimates was then processed using current 4.0/4.1 version of the Interview Research Software and minutes above the 3METs threshold were computed. For MTI data, Matthews cut-point of 760cpm was then used to compute physical activity intensity.<br>*Method*: Methods were tested using IDEEA comparison.<br>*Sync*: Yes.	F: not defined<br>I: MVPA >760cpm for MTI / estimate from the sofware for SP2.<br>T: not defined<br>T: daily physical activities	Pairwise correlations Bland-Altman plots	NA

Yngve et al., 2003	[b]	To define cut-points for estimating physical activity intensity	28 healthy participants (14w/14m)	Oxygen uptake estimation using indirect calorimetry (Cosmed K4, NA, 15s)	MTI 7164 (uniaxial accelerometer, hip/waist and lower back-worn, 15s)	*Setting*: Laboratory *Protocol*: Two walking bouts and one running bout (5min) at different speeds (“normal [walking] pace”, “fast [walking] pace” and "comfortable [running] pace") were completed, once on a 105m indoor track and once on a treadmill.	*Criterion data*: First and last minutes of each stage were ignored then the averaged VO2 values were considered to represent each speed. Activity energy expenditure and METs were also calculated. *Index data*: First and last minutes of each stage were ignored then the averaged activity counts per minute were considered to represent each speed. *Method*: Monitor was tested in different locations and settings. Cut-points were defined using linear regression models. *Sync*: Yes.	F: not defined I: location and setting specific cut-points for MPA and VPA T: not defined T: walking and running activities	ANOVA MANOVA Linear regression analyses Bland-Altman plots	NA

Legend. ANOVA, ANalysis Of Variance; APE, Absolute Percentage Error; ICC, Intraclass Correlation Coefficient; FITT, Frequency Intensity Time Type; LIPA, Light-Intensity Physical Activity; MANOVA, Multivariate ANalysis Of Variance; m, men; MPA, Moderate Physical Activity; MVPA, Moderate-to-Vigorous Physical Activity; NA, not-applicable; NK, not known; R^2, coefficient of determination; rMSE, root Mean Squared Error; SEE, Standard Error of the Estimate; VPA, Vigorous Physical Activity; w, women; yrs, years. [a] not a validation study as such, but proposed a new method based on previously published data. [b] Troaino et al., 2008 is based from 4 studies (Brage et al., 2003, Freedson et al., 1998, Leenders et al., 2001, Yngve et al., 2003) so please also refer to this study for identifying native epidemiological study. [c] several monitors, algorithms and/or methods were tested in this study but only the one considered most relevant is presented here.
